# Reconstruction of complex single-cell trajectories using CellRouter

**DOI:** 10.1038/s41467-018-03214-y

**Published:** 2018-03-01

**Authors:** Edroaldo Lummertz da Rocha, R. Grant Rowe, Vanessa Lundin, Mohan Malleshaiah, Deepak Kumar Jha, Carlos R. Rambo, Hu Li, Trista E. North, James J. Collins, George Q. Daley

**Affiliations:** 1 Stem Cell Transplantation Program, Division of Pediatric Hematology and Oncology, Boston Children’s Hospital and Dana-Farber Cancer Institute, Boston, MA 02115 USA; 2 Department of Biological Chemistry and Molecular Pharmacology, Harvard Medical School, Boston, MA 02115 USA; 3 Harvard Stem Cell Institute, Cambridge, MA 02138 USA; 4 Manton Center for Orphan Disease Research, Boston, MA 02115 USA; 5 Department of Systems Biology, Harvard Medical School, Boston, MA 02115 USA; 6 Present Address: Department of Electrical and Electronic Engineering, Federal University of Santa Catarina, Florianopolis, 88040-900 Brazil; 7 Department of Molecular Pharmacology and Experimental Therapeutics, Mayo Clinic, 200 First Street SW, Rochester, MN 55905 USA; 8 Institute for Medical Engineering & Science, Department of Biological Engineering, and Synthetic Biology Center, Massachusetts Institute of Technology, Broad Institute of MIT and Harvard, Cambridge, MA 02142 USA; 9 Wyss Institute for Biologically Inspired Engineering, Harvard University, Boston, MA 02115 USA; 10 Division of Systems Biology, Montreal Clinical Research Institute, 110 Avenue Des Pins Ouest, Montreal, QC H2W 1R7 Canada

## Abstract

A better understanding of the cell-fate transitions that occur in complex cellular ecosystems in normal development and disease could inform cell engineering efforts and lead to improved therapies. However, a major challenge is to simultaneously identify new cell states, and their transitions, to elucidate the gene expression dynamics governing cell-type diversification. Here, we present CellRouter, a multifaceted single-cell analysis platform that identifies complex cell-state transition trajectories by using flow networks to explore the subpopulation structure of multi-dimensional, single-cell omics data. We demonstrate its versatility by applying CellRouter to single-cell RNA sequencing data sets to reconstruct cell-state transition trajectories during hematopoietic stem and progenitor cell (HSPC) differentiation to the erythroid, myeloid and lymphoid lineages, as well as during re-specification of cell identity by cellular reprogramming of monocytes and B-cells to HSPCs. CellRouter opens previously undescribed paths for in-depth characterization of complex cellular ecosystems and establishment of enhanced cell engineering approaches.

## Abstract (web summary)

Single cell analysis provides insight into cell states and transitions, but to interpret the data, improved algorithms are needed. Here, the authors present CellRouter as a method to analyse single-cell trajectories from RNA-sequencing data, and provide insight into erythroid, myeloid and lymphoid differentiation.

## Introduction

Gene expression profiling has been widely applied to understand regulation of cellular processes in development and disease^1^. However, micro-environmental influences, asynchronous cell behaviors, and molecular stochasticity often leads to pronounced heterogeneity in cell populations, obscuring the dynamic biological principles governing cell-state transitions. Single-cell, high-throughput technologies present an opportunity to characterize these states and their transitions by simultaneously quantifying a large number of parameters at single-cell resolution. However, as cells are destroyed during measurement, data-driven approaches are required to illuminate the dynamics of cellular programs governing fate transitions. To study gene expression dynamics, several algorithms have been developed to organize single cells in pseudo-temporal order based on transcriptomic or proteomic divergence^2–6^. While current algorithms best identify trajectories between the most phenotypically distant cell states, which molecularly are very distinct, they are less robust in reconstructing trajectories from early states towards intermediate or transitory cell states. Limitations include reconstructing linear trajectories (Waterfall, Monocle 1), identifying only a single branch point (Wishbone), or requiring a priori knowledge of the number of branches (Diffusion Pseudotime, DPT). Monocle 2 addresses many of these challenges but is not designed to reconstruct trajectories between any two chosen cell states, which might include transitions from or to rare cell types. Moreover, as they are designed to identify branching trajectories, Wishbone, DPT, and Monocle 2 are less suited to detect convergent differentiation paths, such as during plasmacytoid dendritic cell development from distinct precursor cells^7^.

To overcome these challenges, we developed CellRouter (Supplementary Software 1–4, https://github.com/edroaldo/cellrouter), a general single-cell trajectory detection algorithm capable of exploring the subpopulation structure of single-cell omics data to reconstruct trajectories of complex transitions between cell states. CellRouter requires no a priori knowledge of trajectory structure, such as number of cell fates or branches. CellRouter is a transition-centered trajectory reconstruction algorithm, distinct from the bifurcation-centered algorithms such as Wishbone, DPT, and Monocle 2. While bifurcations occur during lineage diversification, transitions also converge to specific lineages or occur between cell states within branches. CellRouter relaxes the requirement of identifying branching points during cell-fate transitions and implements a flow network algorithm to flexibly reconstruct multi-state transition trajectories. Moreover, CellRouter is independent of dimensionality reduction techniques and can be used, for example, with principal component analysis (PCA), t-stochastic neighbor embedding (t-SNE) or diffusion maps.

CellRouter is a flexible single-cell analysis platform designed to reconstruct single-cell trajectories of complex cell-state transitions. We apply CellRouter to several single-cell RNA-sequencing data sets to provide insight into multi-lineage differentiation from hematopoietic stem and progenitor cells (HSPCs) in snapshot data sets and also during a time-course of mesoderm diversification towards the blood lineage, revealing sequential waves of gene expression changes along differentiation trajectories. Moreover, we provide insight to guide cellular reprogramming by exploring stem cell differentiation data sets as a blueprint to identify reprogramming trajectories and develop new cell engineering strategies. CellRouter integrates subpopulation identification, multi-state trajectories, and gene regulatory networks (GRNs) to provide new insights into cell-state transitions during lineage diversification, convergence, or cell reprogramming.

## Results

### Reconstructing complex single-cell trajectories

**Fig. 1:**
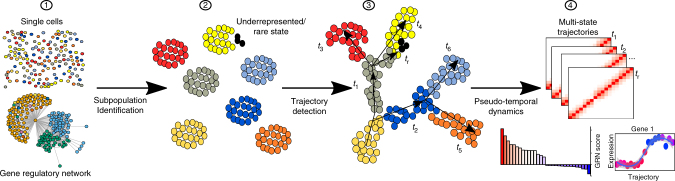
Overview of CellRouter. Step (1) Starting from single cells representing multiple cell states, including stable and in transition, a gene regulatory network was built to identify putative gene regulatory relationships. Step (2) Subpopulations were identified by a combination of learning the structure of the data and community detection algorithms. Step (3) High-resolution multi-state trajectories are identified from any subpopulation to any other, including intermediate and mature cell types. Step (4) Multi-state trajectories illustrate the divergence of single-cell transcriptomes along each trajectory progression. Identification of genes implicated in the dynamic biological process under study, such as differentiation, and identification of regulators driving or mediating cell-fate transitions at the gene and network level

To identify multi-state transition trajectories, CellRouter builds a k-nearest neighbor (kNN) graph from cell−cell relationships in a space of reduced dimensionality (Fig. 1). CellRouter then transforms the kNN graph to represent cell−cell similarities by assigning weights to each edge based on network similarity metrics (e.g., the Jaccard similarity). This approach weakens connections between unrelated cell types and strengthens connections between cells within the same subpopulation, better representing phenotypic relatedness^8^. Next, using community-detection algorithms (e.g., the Louvain method), subpopulations are defined by identifying communities of densely inter-connected cells^8, 9^. Then, CellRouter uses a graph theory approach to solve the minimum cost flow problem and precisely define trajectories between any two subpopulations (*t*_1_, *t*_2_,.., *t*_6_)^10, 11^, including transitions to intermediate states (*t*_1_, *t*_2_) or rare or under-represented cell types or states (*t*_r_) (Fig. 1, Supplementary Note 1, Supplementary Method). Importantly, CellRouter identifies a subset of representative transitioning cells, better accounting for stochastic or regulated cell-to-cell variation. Finally, to account for drop out events in single-cell RNA-seq data, CellRouter explores the local topology of the kNN graph to smoothen the kinetic trends along each trajectory.

### CellRouter identifies transition-specific gene dynamics

**Fig. 2:**
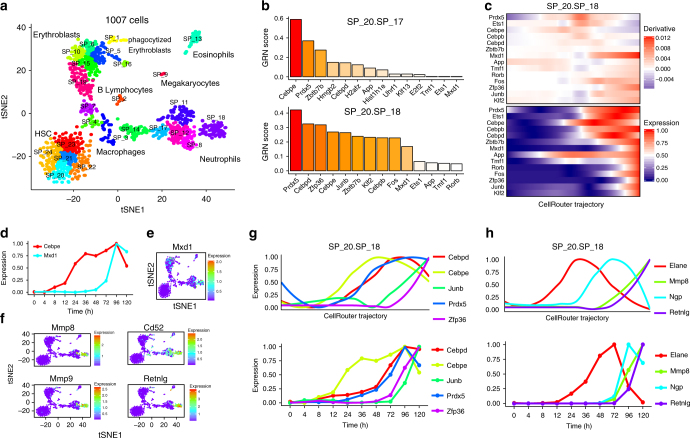
Gene expression dynamics during neutrophil differentiation. **a** t-SNE plot using genes reliably expressed as identified in the original study^12^. **b** Predicted transcriptional regulators during cell-fate transitions from hematopoietic stem cells to neutrophil progenitors (subpopulation 17) and mature neutrophils (subpopulation 18). **c** Transcriptional dynamics from hematopoietic stem cells (subpopulation 20) to mature neutrophils (subpopulation 18). **d** Validation of the developmental timing of *Cebpe* and *Mxd1* by bulk RNA-seq along a time-course of neutrophil differentiation. **e** t-SNE map colored by expression of *Mxd1*. **f** t-SNE maps colored by expression of *Mxd1* predicted target genes during neutrophil differentiation. **g** Kinetic trends of top regulators of transitions from HSCs to subpopulation 18 in **b**, top panel, and validation of these patterns by bulk RNA-seq during a time-course of neutrophil differentiation, bottom panel. **h** Genes known to be important in progenitor and mature neutrophils, top panel, and the validation of their expression dynamics using the time-course of neutrophil differentiation, bottom panel. Accession codes: GSE76983 and GSE84874

We applied CellRouter to a mouse bone marrow single-cell RNA-seq data set (Supplementary Software 1). This data set contains a non-random sampling of bone marrow cells isolated by microdissection and also includes a purified Kit^+^Sca-1^+^Lin^−^CD48^−^CD150^+^ population enriched for hematopoietic stem cells (HSCs)^12^. We identified 24 subpopulations, corresponding to a variety of cell types including HSCs, erythroblasts, megakaryocytes, neutrophils, basophils, macrophages, B-lymphocytes, and other intermediate subpopulations (Fig. 2a). Annotation of cell types was based on the original publication^12^ and gene signatures identified by CellRouter (Supplementary Data 1).

CellRouter ordered single cells along their major branching differentiation trajectories towards erythroblasts and neutrophils. Of note, subpopulation 20, which expresses high levels of *Eef1a1*, was selected as the starting point for trajectory identification (Supplementary Fig. 1a). We used known erythroid and neutrophil genes as positive and negative controls for these differentiation trajectories, with erythroid genes but not neutrophil genes, dynamically regulated during erythroid differentiation. A similar, but opposing pattern, is observed in the neutrophil trajectory (Supplementary Fig. 1b). CellRouter predicted the trajectories as a continuum of cell-state transitions by classifying several subpopulations as the intermediate or transitory states between the HSCs and erythroblasts or neutrophils. We developed a scoring scheme to identify transcriptional regulators by their status of activation and by their predicted target genes, termed GRN score (Methods). In the neutrophil branch, this analysis identified *Cebpe* as a central regulator of neutrophil progenitor GRNs, consistent with its known functional roles in granulopoiesis^13^. Other top-ranked genes identified in this analysis are less well characterized (Fig. 2b, top panel). In mature neutrophils (subpopulation 18), the GRN score for *Mxd1* substantially increases (Fig. 2b, bottom panel). To our knowledge, the role of *Mxd1* in neutrophil differentiation remains largely uncharacterized. In the neutrophil trajectory, *Mxd1* is upregulated in later stages of differentiation compared to *Cebpe* (Fig. 2c, d). According to GRN analyses, *Mxd1* is predicted to regulate *Mmp8*, *Mmp9*, *Retnlg,* and *Cd52* expression, all of which are known neutrophil markers (Fig. 2e, f). Interestingly, *Cebpe* is upregulated early along the trajectory to neutrophils, followed by *Prdx5* and *Cebpd* and then *Junb* and *Zfp36* (Fig. 2c, g, top panel). In addition, we observed a sequence of gene activations characteristic of neutrophil differentiation, which could further distinguish early transitions (characterized by *Elane* expression) from late transitions (*Ngp* expression), a temporal relationship not previously reported. Finally, we observed upregulation of mature makers, such as *Mmp8* and *Retnlg* (Fig. 2h, top panel). To validate these findings, we re-analyzed bulk RNA-seq data over time in a model of temporal neutrophil differentiation^14^. This analysis showed that expression changes identified by CellRouter and the time-course of neutrophil differentiation were highly consistent, demonstrating accuracy of the predicted trajectory (Fig. 2g, h, bottom panels). In addition, the relative timing of changes in gene expression with respect to one another was highly consistent between the predictions and the time-course data.

**Fig. 3:**
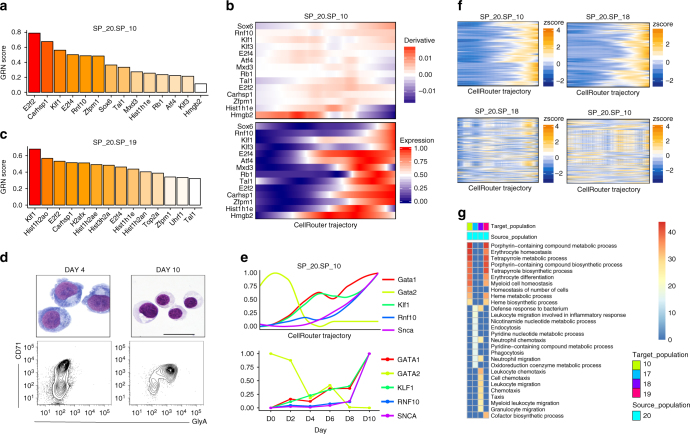
Gene expression dynamics during erythroid differentiation. **a** Predicted transcriptional regulators of transitions from HSCs to erythroblasts (subpopulations 20 to 10). **b** Transcriptional dynamics of top regulators of transitions from HSCs (subpopulation 20) to the erythroblast subpopulation 10. **c** Predicted transcriptional regulators of transitions from HSCs to the erythroblast progenitor subpopulation 19. **d** Staining and representative fluorescence-activated cell sorting (FACS) profiles of human CD34^+^ peripheral blood cells induced to differentiate to erythroid cells (scale bar: 100 μm). **e** Transcriptional dynamics along the CellRouter trajectory of genes involved in mouse and human erythroid differentiation from HSCs to erythroblasts (subpopulations 20 to 10, top panel) and the qPCR validation of these patterns during human erythroid differentiation, bottom panel. **f** Transition-specific gene expression, as shown by the presence of an expression pattern in the top panel and lack of any patterns in the bottom panel. **g** Gene ontology enrichment analysis of genes positively regulated during HSC differentiation (subpopulation 20) to erythroid progenitors and more mature erythroblasts (subpopulations 10 and 19) as well as neutrophil progenitors and mature neutrophils (subpopulations 17 and 18). Expression of each curve was normalized between 0 and 1 to highlight their dynamic patterns. Accession code: GSE76983

In the erythroblast branch, CellRouter predicted candidate genes involved in erythroid differentiation, such as *Rnf10*, as well as their expression dynamics along the pseudo-time (Fig. 3a, b). Moreover, CellRouter also identified putative regulators of transitions to progenitor stages of erythroid differentiation (Fig. 3c). Next, we validated kinetic patterns for selected erythroid genes and *Rnf10* by differentiating human erythrocytes from peripheral blood CD34^+^ cells^15^, and measuring transcripts by quantitative polymerase chain reaction (qPCR) at various time points (Fig. 3d, e). RNF10 interacts with the GATA1-regulated protein SCNA^16^, which is highly correlated with the progression from the HSC subpopulation 20 to the erythroblast subpopulation 10. Moreover, *Gata1* is upregulated earlier than *Scna*, consistent with activation of *Scna* downstream of *Gata1* both in the CellRouter trajectory and during in vitro erythroid differentiation (Fig. 3e and Supplementary Fig. 1c). In addition, using differential expression analysis, we identified five transcription factors—*E2f4*, *H2afx*, *Klf1*, *Tal1,* and *Zfpm1*—which were highly expressed in subpopulation 10 compared to all other subpopulations. Using qPCR, we validated their dynamics during in vitro erythroid differentiation. Experimental measurements confirmed that these genes were activated during terminal differentiation, which is consistent with the CellRouter trajectory (Supplementary Fig. 1d, e).

Next we used genes with increasing expression during differentiation towards erythroblasts (subpopulation 10, marked by highest *Klf1* levels, Supplementary Fig. 1a, Supplementary Data 1) and mature neutrophils to visualize how these genes change along the opposite trajectory. While a clear pattern was observed for transition-specific genes in their respective trajectories, no pattern was observed in the unrelated trajectory (Fig. 3f). Gene Ontology analysis of transition-specific genes towards erythroblast progenitors and more mature cell states (subpopulations 19 and 10, respectively) and towards neutrophil progenitors and mature neutrophils (subpopulations 17 and 18, respectively) showed that genes enriched in the mature cell states are consistent with the predicted cell identity (Fig. 3g, Supplementary Data 2). These results demonstrate that CellRouter identifies transition-specific gene expression dynamics and regulatory nodes during lineage diversification from HSPCs in the mouse bone marrow.

### CellRouter identifies multi-lineage single-cell trajectories

**Fig. 4:**
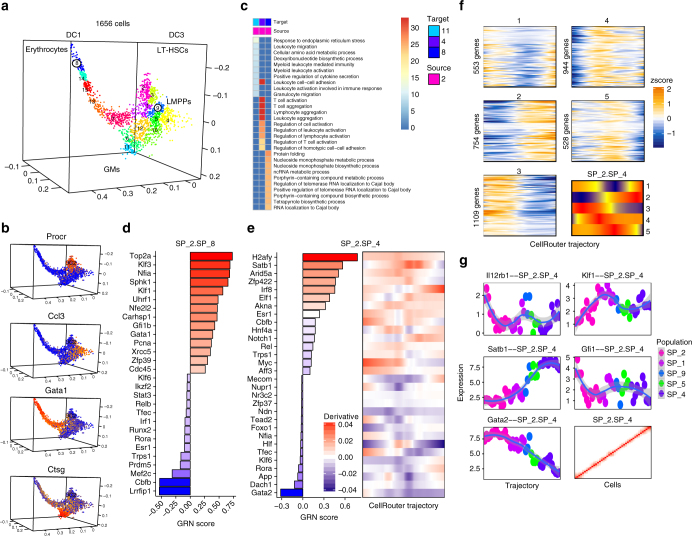
Multi-lineage hematopoietic stem cell differentiation. **a** Three-dimensional diffusion components analysis with subpopulation structure identified by CellRouter. **b** Identification of lineages based on expression of known marker genes with *Procr* expressed in HSCs and *Gata1*, *Ccl3*, and *Ctsg* expressed in the erythroid, lymphoid, and granulocyte-macrophage lineages, respectively. **c** Gene ontology analysis using genes dynamically upregulated in selected differentiation trajectories from HSCs to the erythrocytes (subpopulation 8), granulocyte/macrophage progenitors (subpopulation 11), and lymphoid progenitors (subpopulation 4). **d** Predicted regulators of transitions to the erythroid lineage (subpopulation 8). **e** Predicted transcriptional regulators of lymphoid development from HSCs (left panel) and their pseudo-temporal regulation (right panel). **f** Clustering of gene expression trends during lymphoid development from HSCs into five expression patterns, with a summary of the patterns in each cluster shown in the bottom right panel. **g** Representative genes in transcriptional clusters from **f** and the Jaccard similarity matrix of the lymphoid trajectory demonstrating the gradual divergence of single-cell transcriptomes during differentiation (bottom right panel). Accession code: GSE81682

We next tested CellRouter on the BloodNet data set, which contains a spectrum of stem, progenitor, and differentiated cells^17^ (Supplementary Software 2). This analysis highlighted the flexibility of CellRouter to identify differentiation trajectories from any dimensionality reduction technique and find a refined cell-state transition structure. By building a kNN graph from three-dimensional diffusion components from the original publication, CellRouter identified 15 subpopulations (Fig. 4a). Subpopulation 2 was enriched for long-term HSCs and it was used as the starting population for the identification of developmental trajectories toward hematopoietic ontogeny. Subpopulations 8, 11, and 4 were enriched for erythrocytes, granulocyte-macrophages (GM), and lymphoid multipotent progenitors (LMPP), respectively (Fig. 4b, Supplementary Data 3). We used erythroid, GM, and LMPP genes as positive and negative controls of these differentiation trajectories, with erythroid genes (*Klf1*, *Gata1*, *Gfi1b*) but not GM (*Ctsg*, *Gfi1*) or LMPP (*Ccl3*) genes dynamically regulated during erythroid differentiation. *Ccl3* correlated with transitions to LMPPs but not with the GM or erythrocyte fates. Finally, GM genes were expressed during GM differentiation but not erythroid or LMPP differentiation, suggesting that CellRouter correctly identified multi-lineage differentiation trajectories (Supplementary Fig. 2a). Gene expression along each trajectory was highly enriched for lineage-specific biological processes such as “phorphyrin metabolic processes”, “granulocyte migration”, and “T-cell activation”, demonstrating that CellRouter can capture transition-specific gene expression dynamics during HSC differentiation to multiple lineages (Fig. 4c, Supplementary Data 4).

Next we used CellRouter to understand two potential regulatory mechanisms: (1) upregulation of transcription factors and their predicted target genes to capture direct induction of lineage-specific transcriptional programs; and (2) downregulation of transcription factors leading to increased expression of target genes, to capture de-repression of lineage specifying genes during differentiation. In both models, target genes were upregulated during differentiation, but their regulators could be upregulated (in red) or downregulated (in blue) (Fig. 4d, e). Consistently, *Klf1*, *Gata1*, *Gfi1b*, and *Nfia* were among the top genes driving the erythroid fate (subpopulations 2 to 8 transition). In LMPPs (subpopulations 2 to 4 transition), *Satb1*, *Irf8*, *Notch1*, and *Esr1* were among the top predicted genes, confirming previous reports^18–20^ (Fig. 4e, left panel). Temporally, *Satb1* was upregulated early and its expression peaked in an intermediate position along the trajectory, consistent with its role during commitment of lymphoid progenitor cells^19^. *Irf8* and *Esr1* were transiently downregulated, with more pronounced changes in *Irf8* expression occurring later along the lymphoid progenitor trajectory (Fig. 4e, right panel).

It has been reported that *Notch1* promotes self-renewal over differentiation in stem cells, and lymphoid over myeloid lineage fates, consistent with our analysis^20^. Transition-specific regulators of intermediate subpopulations for each lineage suggested the existence of mixed states primed to GMs and lymphoid progenitors, as indicated by shared regulatory genes such as *Satb1*, *Arid5a*, *Lmo4*, and *Elf1* (Supplementary Fig. 2b–d). Finally, we clustered gene expression dynamics from long-term HSCs to LMPPs to characterize waves of transcriptional changes along this trajectory (subpopulations 2 to 4) (Fig. 4f, Supplementary Fig. 2e, Supplementary Data 5). Representative genes in each cluster are shown in Fig. 4g. This temporal information is crucial to understand the dynamics of regulatory interactions underlying cell-state transitions: genes activated earlier along differentiation trajectories are potential regulators of this dynamic biological process, while genes activated late are likely involved in cell-fate commitment.

### Insights for reprogramming blood cell types to HSPCs

**Fig. 5:**
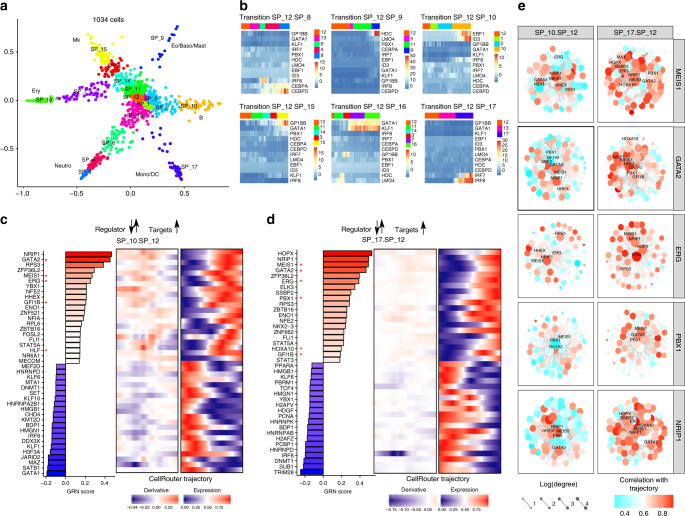
Application of CellRouter to gain insight for cell reprogramming. **a** STEMNET dimensionality reduction with subpopulations identified by CellRouter and cell types annotated based on the original study. **b** Positive and negative controls for lineage-specific differentiation trajectories. **c** Left panel: predicted candidates to reprogram B-cells (subpopulation 10) to HSPCs (subpopulation 12) based on overexpression (genes in red) or knockdown (genes in blue) of indicated transcriptional regulators. Middle panel: Derivative of the gene expression dynamics for each regulator. Right panel: Kinetic profile of each regulator along the reprogramming trajectory from B-cells to HSPCs. **d** Left panel: predicted candidates to reprogram mono/DCs (subpopulation 17) to HSPCs (subpopulation 12) based on overexpression (genes in red) or knockdown (genes in blue) of indicated transcriptional regulators. Middle panel: Derivative of the gene expression dynamics for each regulator. Right panel: Kinetic profile of each regulator along the reprogramming trajectory from mono/DCs to HSPCs. **e** Regulator-centered subnetworks. * indicates genes previously used to convert or reprogram a starting population to blood progenitors or HSPCs. Accession code: GSE75478

CellRouter’s ability to reconstruct cell-state transition trajectories by exploring subpopulation structure provides flexibility to identify not only differentiation trajectories, but also predict cell reprogramming trajectories. To demonstrate this, we applied CellRouter to human single-cell RNA-seq profiling HSPCs differentiating to six different lineages^21^ (Fig. 5a). We used STEMNET for dimensionality reduction^21^ and applied CellRouter to first identify lineage-specific differentiation trajectories (Supplementary Software 3). We annotated subpopulations as “Endpoint” or “Stem Cell” based on the original study^21^ and selected subpopulation 12, which contains stem cells, as the starting point for trajectory reconstruction (Supplementary Fig. 3a). Also based on the original study^21^, we used genes important for each transition as positive controls. As negative controls, we used genes implicated in unrelated differentiation trajectories. *GATA1*/*KLF1* were used as erythrocyte (Ery) progenitor markers, *GP1BB*/*PBX1* as megakaryocyte (Mk) progenitor genes, *HDC*/*LMO4* as eosino/basophil/mast (Eo/Baso/Mast) cell progenitor markers, *CEBPA*/*CEBPD* as neutrophil (Neutro) progenitor genes, *IRF7*/*IRF8* as monocyte/dendritic cell (mono/DCs) progenitor genes, and *EBF1*/*ID3* as pre-B-cell genes (B) (Fig. 5b, Supplementary Fig. 3b). These analyses confirmed that CellRouter identifies lineage-specific transition trajectories to six different lineages.

Next, we used CellRouter to reconstruct reprogramming trajectories from B-cells and mono/DCs toward HSPCs (Supplementary Software 4). We incorporated GRN analysis to define two regulatory mechanisms: (1) upregulation of transcriptional regulators and their predicted target genes, to capture direct induction of HSPC-specific transcriptional programs; and (2) downregulation of transcription factors leading to increased expression of target genes (i.e., de-repression), to identify genes that must be knocked down in the starting population to induce the HSPC fate. In both models, target genes were upregulated during reprogramming, while their regulators were upregulated or downregulated (Fig. 5c, d). Interestingly, many genes important for HSPC identity were identified, including several described as mediating cell-fate conversions within the hematopoietic system. For example, CellRouter identified *Hlf*, *Pbx1*, and *Meis1*, previously used in combination with other genes to convert mouse B-cells to HSC-like cells^22^; and *Gata2* and *Gfi1b*, previously used in combination with *cFos* and *Etv6* to reprogram murine fibroblasts to blood progenitors^23^. Genes predicted by CellRouter and validated in published reprogramming strategies^22–25^ to generate blood progenitors or HSPCs are highlighted in Fig. 5c, d.

In our previous work, we used libraries of transcription factors to convert human induced pluripotent stem cell (iPSC)-derived blood progenitors to HSPCs^24, 25^. In one study, in vitro screening of a library of nine transcription factors identified five transcriptional regulators that conferred bipotent lineage potential and engraftment in immunodeficient mice^24^. More recently, we used a library of 26 transcription factors to generate engraftable multipotent HSC-like cells from human pluripotent stem cells^25^. Among these transcription factor libraries, including the ones used to respecify murine cells^22, 23^ (67 genes in total, Supplementary Data 6), CellRouter identified 11 of these factors and predicted additional candidates, some of which have known roles in HSC biology (for example, *MECOM*). These engineered HSPCs are still transcriptionally distinct from cord-blood HSPCs^25^. As the libraries of genes used for reprogramming were identified mostly using bulk transcriptomics and literature mining, which might not be the most critical factors to establish bona-fide HSC GRNs in the starting cell population, our methodology provides opportunities to develop new cell engineering strategies by first identifying better gene sets for reprogramming. Similarly, by identifying epigenetic barriers that might be impairing the reprogramming genes to induce HSC gene expression programs, or by exploring time-dependent expression of reprogramming genes and the kinetic patterns of gene expression changes along the reprogramming trajectory, we may better drive the production of functional HSCs.

Interestingly, we observed distinct sets of candidate genes for knockdown in mono/DCs or B-cells. Specifically, we observed that for converting B-cells to HSPCs, our candidate gene list includes chromatin regulators such as *DNMT1*, *H3F3A*, *JARID2*, and *KMT2D* (Fig. 5c), while to convert Mono/DCs into HSPCs, knockdown of *DNMT1*, *H2AFZ*, *TRIM28*, *KMT2D*, and *H2AFV* would be required (Fig. 5d). Regulator-centered subnetworks for selected genes previously used to engineer HSC-like cells showed that these transcriptional regulators form an interconnected regulatory circuitry (Fig. 5e).

Barring *DNMT1*, all other chromatin modifiers that we identified are non-redundant, consistent with a specific epigenetic state in each of the starting cell populations; therefore, depending on the starting epigenetic state, we predict that each starting cell type would require knockdown of a unique set of chromatin modifiers^26^. CellRouter predicts that during these cell-state transitions, these chromatin modulators suppress expression of HSPC genes (Supplementary Fig. 3c). In addition, previous attempts to generate HSC-like cells did not explore the kinetic component of gene expression during the cell engineering, and instead relied on simultaneously inducing the expression of a library of transcription factors. CellRouter provides a rational strategy to identify the (pseudo)timing at which combinations of genes should be overexpressed or downregulated to potentially enhance cell-fate conversions. For example, to convert B-cells to HSPCs, *NFE2* and *FLI1* could be overexpressed early, followed by *NRIP1*, *ERG* and *HLF* and then *GATA2* and *MEIS1*, as these genes present distinct kinetic patterns along the reprogramming trajectory from B-cells to HSPCs (Fig. 5c). This information is crucial to understand the dynamics of regulatory interactions underlying cell-fate reprogramming: genes activated earlier along reprogramming trajectories are initial mediators of this dynamic biological process, while genes activated late are likely involved in establishing or reinforcing the identity of the target cell type. Overall, CellRouter allows for identifying candidate genes in an unbiased but rational manner for cell-engineering purposes.

### Comparison of CellRouter to other algorithms

**Fig. 6:**
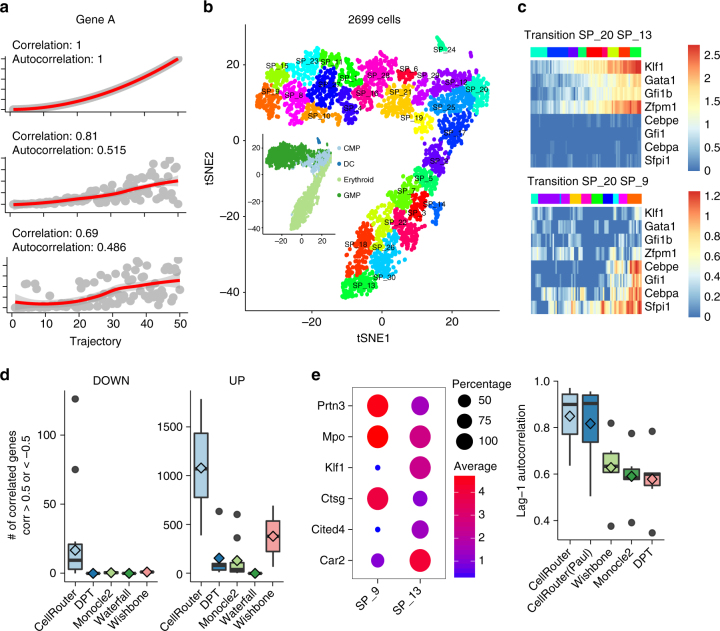
Benchmarking CellRouter to other algorithms. **a** Simulated curves representing gene expression dynamics of a selected gene along differentiation trajectories demonstrating the relationship between correlation and autocorrelation with the trajectory. **b** t-SNE map of myeloid progenitor cells annotated by subpopulations identified by CellRouter. Inset shows broad cell type annotations for each population. **c** Positive and negative controls of erythrocyte and GMP differentiation. **d** Number of genes up- or downregulated significantly correlated with the trajectories identified by each trajectory detection algorithm. **e** Left panel: selected markers of granulocyte/monocyte progenitors and erythrocytes. Size is proportional to the percent of cells expressing the markers in each subpopulation. Color is proportional to mean expression of the markers in each subpopulation. Right panel: lag-1 autocorrelation of marker genes on the right panel along the GMP and erythrocyte differentiation trajectories identified by each trajectory detection algorithm. Accession code: GSE72857

Finally, we compared CellRouter to the established algorithms Monocle 2, DPT, Wishbone, and Waterfall using mouse myeloid progenitor transcriptomes from the data sets generated by Paul et al.^27^ and Olsson et. al.^13^ (Fig. 6, Supplementary Fig. 4a–c, Supplementary Note 2). Unlike CellRouter, previously published methods do not construct an explicit differentiation path to/from multiple cell states (which may also converge to a particular cell type). Instead, Wishbone and DPT identify branches by analyzing patterns that diverge from a linear trajectory, and Monocle 2 constructs an explicit tree^28^. A common feature of most or all of the existing methods, thus far, is that they assign a pseudotime to each cell, “compressing” cells into a single branch-specific trajectory, introducing “noise” to the analysis. In contrast, CellRouter implements an optimization algorithm, based on flow networks, to identify a subset of cells connecting a starting cell to another cell in the target population, which leads to high resolution trajectories, with smoother gene expression dynamics (Figs. 1 and 6a). Therefore, we hypothesized that when we choose a subset of cells connecting any initial subpopulation to a target subpopulation, CellRouter would identify differentiation trajectories with smoother gene expression dynamics compared to other algorithms, potentially uncovering additional genes involved in the transition that are undetectable by previous methods (Fig. 6a). To test this, we used two metrics to compare CellRouter to previously described algorithms. First, the number of genes significantly correlated to each transition or branch-specific trajectory and second, the lag-1 autocorrelation of selected marker genes. Higher numbers of correlated genes and higher lag-1 autocorrelations imply smoother gene expression dynamics, reflecting the quality of the trajectory reconstructed by each algorithm (Fig. 6a). We applied CellRouter to identify differentiation trajectories from common myeloid progenitors (CMPs, subpopulation 20) to intermediate and mature cell states in the megakaryocyte/erythrocyte progenitor (MEPs) and granulocyte/monocyte progenitor (GMPs) branches in the data set generated by Paul et al.^27^ (Fig. 6b). We used genes known to be important in specifying the MEP and GMP branches as positive and negative controls for the two selected differentiation trajectories to these branches^27^ (Fig. 6c), confirming that CellRouter reconstructs transition-specific trajectories.

We first calculated the number of significantly up- or downregulated genes along differentiation trajectories identified by CellRouter, Monocle 2, DPT, Wishbone, and Waterfall (Fig. 6d). Next, based on the prior study by Paul et al.^27^, we selected known markers of each branch, such as *Klf1*, *Car2*, and *Cited4* in the erythroid branch, and *Mpo*, *Prtn3*, and *Ctsg* in the GMP branch, and calculated the lag-1 autocorrelation of these markers along the respective trajectories, from subpopulation 20 (CMP) to subpopulation 13 (erythroid branch) or to subpopulation 9 (GMP branch) (Fig. 6e). We also applied CellRouter either using the subpopulation structure determined by CellRouter in Fig. 6b or clusters identified by Paul et al.^27^ to evaluate the effect of varying subpopulation structure (Supplementary Fig. 4d). These analyses showed that trajectories identified by CellRouter are smoother compared to other algorithms, reflecting the quality of CellRouter’s trajectories (Fig. 6e, right panel). We also evaluated the effect of changes in subpopulation structure (Fig. 6e, Supplementary Fig. 4d–f), performed a comparison to other methods in two synthetic data sets generated by Splatter^29^ (Supplementary Fig. 4g,h) and assessed the robustness of CellRouter’s trajectories to subsampling (Supplementary Fig. 4i). Together these analyses showed that trajectories identified by CellRouter are robust to subpopulation structure and subsampling, and also outperform the previously described algorithms by all of these metrics.

Moreover, we performed a side-by-side comparison of CellRouter to Monocle 2 using the Olsson data set^13^, which contains an underrepresented bifurcation of the megakaryocyte/erythrocyte lineages (Supplementary Fig. 5). While CellRouter identified differentiation trajectories from HSPCs to megakaryocytes or erythrocytes (subpopulations 19 and 5, respectively), Monocle 2 did not distinguish the megakaryocyte/erythrocyte lineages, and assigned them to the same branch (Supplementary Fig. 5a–e, Supplementary Figs. 7 and 8, Supplementary Data 7, 8 in Supplementary Note 3**)**. Finally, we assessed how the choice of dimensionality reduction technique affects trajectories reconstructed by CellRouter, and analyzed the feasibility of applying CellRouter to convergent differentiation paths (Supplementary Note 4). These analyses demonstrated that trajectories identified by CellRouter are robust to the choice of dimensionality reduction technique and also capable of analyzing cell populations undergoing convergent differentiation processes. The ability of CellRouter to explore subpopulation structure to identify multi-state transition trajectories provides enhanced resolution to study the dynamics of cell-fate transitions. Application of CellRouter to other studies demonstrates its versatility in analyzing both snapshot and time-course single-cell transcriptomics data sets (Supplementary Figs. 9 and 10, Supplementary Data 9–11, Supplementary Note 3).

## Discussion

CellRouter introduces the concept of subpopulation-awareness to identify cellular trajectories and gene expression dynamics between any subpopulations using a network representation of cell−cell relationships learned from a low-dimensional embedding. This network and the underlying subpopulation structure are used as a map of cell-fate transitions. Utilizing concepts from flow networks and solving the multi-source/multi-target minimum cost flow problem to optimally connect cells in different locations of this map (subpopulations), CellRouter allows the study of expression dynamics in bifurcating or convergent differentiation paths, as well as during cell-fate engineering, in many different branches or within the same branch. Integration with GRNs enhances trajectory analysis by allowing identification of putative regulators for cell-state specification during lineage differentiation or reprogramming. Moreover, cell-state transition trajectories involving multiple branches present in the subpopulation structure can be analyzed, simultaneously, without any assumptions related to the structure of the trajectories such as the number of branches, or removal of unrelated cell types.

CellRouter allowed us to accurately reconstruct multi-lineage differentiation dynamics during erythroid, myeloid, and lymphoid differentiation from HSPCs. Specifically, it allowed us to study neutrophil differentiation dynamics and implicate additional genes in erythroid differentiation. Existing attempts to convert or reprogram one cell type into another have been largely focused on the simultaneous overexpression of a defined set of transcription factors in the starting cell population. However, in the case of HSPCs, epigenetic barriers may limit the efficiency of cell-fate conversion and potentially impair the acquisition of multi-lineage differentiation potential. In addition to identifying and removing the epigenetic barriers, exploring the kinetic component of gene expression during cell-state transitions by mimicking, in vitro, the sequence of gene activation and repression events along the reprogramming trajectory could further enhance cell-fate engineering. CellRouter achieves both of the above and thus provides a rational strategy to develop optimized cell engineering protocols, which may require the time-sensitive addition and/or inhibition of morphogens, small molecules, cytokines, and transcription factor overexpression. While thorough characterization of the potential for each of the genes identified by CellRouter to lead to the generation of engraftable HSC-like cells with long-term, multilineage potential extends beyond the scope of this manuscript, it warrants future investigations.

Finally, characterization of complex cellular ecosystems from single-cell omics data will require integrated computational platforms to discover both rare cell types as well as multi-state transition trajectories. These are essential to uncover the dynamics of cell-fate transitions conferring cellular diversity to a tissue ecosystem. CellRouter addresses many of these challenges by integrating GRNs with multi-state transition trajectories and scoring schemes to capture transition-specific gene expression and putative regulatory interactions. Moving beyond single-cell transcriptomics, we envision future application of CellRouter to single-cell chromatin accessibility, DNA methylation, and mass cytometry data, thereby providing a valuable multifaceted single-cell analysis platform to the research community for use in a broad array of cell and tissue analyses.

## Methods

### CellRouter algorithm

To identify multi-state trajectories, CellRouter makes the following assumptions: first, as cells evolve asynchronously, a single-cell-based snapshot of a primary tissue captures the entire process of cell-fate diversification within that tissue, such as stem/progenitor cell differentiation, oncogenic transformation or tumor progression; second, within a population, a continuum of phenotypically distinct subpopulations exists; and third, state transitions are continuous with molecular hallmarks activated or silenced in a progressive manner. CellRouter functions in three steps. First, is the reconstruction of GRNs using a modified version of our previously developed algorithm, the context likelihood of relatedness (CLR)^9, 30^, and subpopulation identification; second, is the reconstruction of multi-state trajectories between subpopulations and third, is the downstream analysis based on an integration of GRNs with multi-state trajectories.

As cells progress through a dynamic process, such as differentiation, oncogenic transformation or response to drugs, changes in cellular states occur that are reflected by molecular changes generating both phenotypically stable cell types as well as a continuum of cell states transitioning between phenotypic landscapes. Major challenges involve identifying rare and abundant cell types and their transcriptional dynamics during cell-fate transitions towards multiple cell states, including progenitor and mature cell types, in many distinct lineages or branches.

CellRouter takes a distinct approach compared to previously published algorithms by introducing the concept of subpopulation-aware trajectory identification where transitions between any subpopulations can be studied, regardless of how complex or how many branches might exist in the data set. The advantage of this strategy is twofold. This is first, for the characterization of subpopulation structure and identification of cell types or states and second, identification of multi-state trajectories without any assumptions about branching processes in the same computational platform. To achieve these goals, CellRouter is formulated based on a graph-based clustering algorithm, for subpopulation identification, and a graph theory framework known as flow network, where the identification of multi-state trajectories is formulated as an optimization problem.

### kNN graph construction and subpopulation identification

As the flow network approach requires a graph, the very first step in CellRouter is to carefully build a graph from the single-cell data. This task is performed in three steps. The first step is dimensionality reduction; the second, construction of a kNN graph in the space of reduced dimensions, and the third, is transforming the kNN graph to encode cell−cell similarities. CellRouter takes as input a matrix *M* representing cell−cell relationships in an embedding space and therefore, any dimensionality reduction technique can be used, such as PCA, t-SNE, diffusion maps or others. Indeed, we tested CellRouter with t-SNE and diffusion components. Dimensionality reduction helps to remove short-circuits from the graph, which are edges connecting cells not developmentally related but are connected likely due to measurement noise. The kNN graph is then built from cell−cell relationships in the embedding space, where a cell is connected to their k most similar neighbors using Euclidean distance. Given this graph, we transform cell−cell distances into cell−cell similarities using network similarity metrics such as the Jaccard similarity. For example, the Jaccard similarity will strengthen connections between phenotypically related cells and weaken connections between cells belonging to another subpopulation or due to noise, and therefore, better encoding subpopulation structure and transitional cell states in the graph. The advantage of this transformation is twofold: first, as the graph encodes cell−cell similarities, methods to find community structure in networks can be used to identify subpopulations and second, trajectory detection can be formulated as an optimization procedure to maximize similarity. By doing this, CellRouter can explore the underlying subpopulation structure of the data to find multi-state trajectories with subpopulation resolution.

### Flow network

Mathematically a network is a finite collection of nodes, connected by a finite collection of edges. By convention, a node cannot be connected to itself but many edges can connect a given pair of nodes. We will be working mostly with connected networks, but disconnected networks are also possible. The underlying structure of a flow network is a directed graph where vertices are network nodes and edges represent connections between nodes. Formally, a flow network is defined by the quadruple *N* = (*G*, *s*, *t*, *c*), where *G*(*V*, *E*) is a directed graph of *v* vertices in *V* and *e* edges in *E*, with $E \subseteq V \ast V$, *s* and *t* are auxiliary nodes representing the source and sink of the network, respectively, and *c* is a positive capacity associated to each edge in the graph. Given these basic definitions, we generalize the one-source one-sink framework to solve a multiple-source, multiple-sink problem by introducing the following transformations, which are widely used in optimization theory. Given a collection of nodes to be used as sources *S* and sinks *T*, the previous definitions can be updated as:

${{V}}\prime = {{V}} \cup \left\{ {{{s,t}}} \right\}$, where *s* and *t* are auxiliary nodes used to transform a single-source, single-sink problem into a multiple-source, multiple-sink problem.

${{E}}\prime = {{E}} \cup \left( {{{s,i}}} \right)_{\forall i \in S} \cup \left( {{{t,i}}} \right)_{\forall i \in T}$, connecting sources and sinks to the auxiliary nodes *s* and *t*.

The following optimization problem is solved:

$$\mathrm{min}_f\left( {\mathop {\sum}\limits_{i \in V\prime ,j \in V\prime } { - \mathrm{log}\left( {c_{i,j}} \right) \ast f_{i,j}} } \right)$$

A function *f*=*E* → *R*^+^ is a flow over the network if the following requirements are fulfilled:

$$0 \le {{f}}_{{{ij}}} \le {{c}}_{{{ij}}},\forall \left( {{{i,j}}} \right) \in {{E}}\prime$$

$$\mathop {\sum}\limits_{j \in V\prime } {f_{ij}} - \mathop {\sum}\limits_{j \in V\prime } {f_{ji}} = 0,\forall i \in {{V}}\prime - \left\{ {{{s,t}}} \right\}$$

$$\mathop {\sum}\limits_{i \in S} {f_{{{si}}}} - \mathop {\sum}\limits_{i \in T} {f_{{{it}}}} = 0$$

where *f*_*ij*_ is the flow from node *i* to node *j*. The minimum cost flow problem is to find the flow *f* with maximum value at the lowest cost. To that end, the cost associated to each edge is defined by

$${\mathrm{costs}} = - {\mathrm{log}}\left( {c_{{{i,j}}}} \right)$$

Therefore, minimizing the costs will give preference to high-capacity paths through the graph. Now, if we imagine a graph where each node represents a single-cell and each edge connects cells phenotypically similar, whose weights quantify such similarities, the entire graph will encode cell−cell similarities and heterogeneities. In this scenario, the minimum cost flow problem will find paths connecting source(s) to sink(s) that maximize cell−cell similarities. We call these paths trajectories, where sources(s) are starting cell subpopulations, such as stem cells, and sinks or targets, are their differentiated progeny or progenitor-like states.

### CellRouter trajectories

As briefly described in the previous section, and explained in more detail in this section, the flow network algorithm finds several paths connecting sources to sinks in a kNN graph encoding cell−cell similarities. The flow in the resulting flow network is the maximum achievable with the lowest possible cost, meaning that similarity was maximized. Each path has three properties: a total cost, which is the sum of the cost of each edge; a total flow, which is the sum of the flow of each edge; and a length, which is the number of edges in a path. After the algorithm has finished, each path has a total flow, that it is sum of the flow in each component edge of the path. We then rank paths by normalizing the path total flow by the path length and select the top scoring path as a representative trajectory.

By user choice, paths can be ranked by cost, total flow or length, and the top ranked path will be considered as representative of the trajectory associated with a particular transition.

### Analysis of gene expression dynamics

Once trajectories are identified, CellRouter rank genes for each trajectory according to their correlation (such as Pearson or Spearman, user defined) with the trajectory progression to find genes directly regulated during the biological processes taking place, such as differentiation. In addition, to find more complex gene expression kinetics, CellRouter smooths the actual transcriptional profiles by fitting a smooth spline curve for each gene, for each trajectory. Standardizing these curves allows for efficient K-medoid clustering for all genes regulated along each trajectory. Pairwise distances between genes *x* and *y *are calculated as^2^:

$$d({{x,y}}) = 1 - \frac{{c_{{{x,y}}}}}{2}$$

where *c*_*x*,*y*_ is the Pearson correlation coefficient of the smooth curves representing the expression dynamics of genes *x* and *y*. Clusters correspond to genes with similar gene expression dynamics. Clustering based on smooth curves produce more coherent clusters with refined kinetics and then allowing the identification of more complex gene expression patterns. K-medoid clustering was performed on the smoothed curves after log-transformation and standardization using the PAM package in R. Gene expression dynamics along each CellRouter trajectory were clustered using N clusters that are user-defined, to capture high-resolution changes in expression kinetics. Moreover, these fitted kinetic trends are used to investigate the relative timing of gene expression changes along each differentiation trajectory. To make these patterns more evident, curves corresponding to kinetic profiles of each gene are rescaled between 0 and 1.

### GRN score

As the intrinsic heterogeneity (both stochastic and regulated cell-to-cell variation) of single-cell expression profiles can be understood as perturbations, predictions of regulator−target relationships directly from single-cell data are possible^31^. We developed a scoring scheme to rank transcriptional regulators based on their correlation with the trajectory progression, the correlation of their predicted targets, and the extent to which target genes are regulated during a particular trajectory:

$$\mathrm{GRN}_{i,j} = c_{i,j} \ast m_{t,j} \ast n_j$$

where GRN_*i*,*j*_ is the GRN score for regulator *i* along trajectory *j*, *c*_*i*,*j*_ is the Spearman’s rank correlation of transcriptional regulator *i* with the trajectory *j*, *m*_*t*,*j*_ is the mean correlation of predicted targets of gene *i* regulated along trajectory *j* and *n*_*j*_ is the number of predicted targets regulated along trajectory *j*. Formally, the Spearman’s rank correlation of the gene expression dynamics ***X*** along pseudo-time ***Y*** of regulator *i* and trajectory *j*, respectively, is defined by:

$$r_s = \rho _{rg_X,rg_Y} = \frac{{\mathrm{cov}\left( {rg_X,rg_Y} \right)}}{{\sigma _{rg_X}\sigma _{rg_Y}}}$$

***X*** and ***Y*** are converted to the rank variables *rg*_*X*_ and *rg*_*Y*_, *ρ* is the Pearson correlation coefficient of the ranked variables, cov denotes the covariance, and *σ* is the standard deviation of the respective variables. This equation is calculated by the R function cor.test().

We reasoned that if a regulator is well correlated with a differentiation trajectory, it is potentially involved in differentiation (parameter *c*_*i*,*j*_). Moreover, if its predicted target genes are also well correlated with the differentiation trajectory, it is more likely that the regulator is important (parameter *m*_*i*,*j*_). However, it may happen that the regulator only regulates a few genes that are well correlated to the trajectory and the mean correlation of its targets is high. On the other hand, a regulator that has several target genes dynamically regulated during differentiation should score higher than a regulator that has only a few genes well correlated with the trajectory. This is the motivation to include the term *n*_*j*_ in the equation.

### Selecting sources and targets

CellRouter selects one source and one target cell for each pairwise cell-state transition, which are not randomly selected. CellRouter selects as source and target cells the cells that are more distant in the kNN graph by computing all shortest paths connecting each cell in the source population to the target population. Cells at the beginning and ending of the longest path are selected as source or target cells, such that CellRouter can reconstruct a trajectory between the potentially extreme points of each transition.

### Assessing accuracy of trajectories

We reasoned that a properly reconstructed differentiation trajectory would generate smooth gene expression dynamics, as the ordering reflects developmental divergence from early to late stages of differentiation. This is reflected in how well a gene correlates with the progression along differentiation (as determined by the trajectory) and also the autocorrelation of a gene dynamics with lagged values of its transcriptional kinetics. CellRouter identifies genes correlated with each transition-specific trajectory using the Spearman rank correlation and the lag-1 autocorrelation to quantify how smooth gene expression dynamics is along differentiation trajectories. A time series shows autocorrelation if there is a correlation between the lagged values of the time series. Suppose we have a time-series *x*_1_,*x*_2_…*x*_*n*_, where *x*_*t*_ denotes an observation at time *t*. The lag-1 autocorrelation measures the linear relationship between *x*_*t*_ and *x*_*t*−1_ for all time *t*, which is defined as:

$$c_t = \frac{1}{n}\mathop {\sum}\limits_{s = \mathrm{max}\left( {1, - t} \right)}^{\mathrm{min}\left( {n - t,n} \right)} {\left[ {X_{s + t} - \bar X} \right]\left[ {X_s - \bar X} \right]}$$

$$r_t = \frac{{c_t}}{{c_0}}$$

where *c*_*t*_ is the autocovariance and *r*_*t*_ is the autocorrelation. As gene expression along each trajectory/pseudotime can be seen as a time series, we used this metric to estimate the quality of CellRouter's trajectories and compare it to previously reported algorithms. The higher the autocorrelation, smoother gene expression dynamics based on the ordering of single-cells identified by the trajectory detection algorithm. To test this, based on the original study by Paul et al., we selected three GMP markers (*Mpo*, *Ctsg*, and *Prtn3*) and three erythroid markers (*Klf1*, *Car2*, and *Cited4*) and computed the autocorrelation coefficient of these genes in the GMP and erythroid trajectories identified by CellRouter, Monocle 2, DPT, Wishbone, and Waterfall. Moreover, for each trajectory detection algorithm, we calculated the number of significantly correlated genes along trajectories identified by each method.

### Data sets analyzed

Processed data sets were downloaded from each original publication and used as provided by the authors to increase comparability with previous analysis^12, 21^. These data sets have already excluded low-quality cells and filtered out not-informative genes. The following sections provide details about the analysis of each data set. For each data set, genes with zero variance were removed. For dimensionality reduction, we used the same gene sets identified by the authors of the original publications in their analyses.

### Analysis of bone marrow cells

We downloaded bone marrow single-cell RNA-seq data from the accession number GSE76983. We then performed t-SNE analysis using StemID to increase comparability with the original publication (using 2416 genes reliably expressed). These t-SNE coordinates were used as input to CellRouter to build a kNN graph. We set *k* = 5 for subpopulation identification and *k* = 10 for trajectory analysis. In this data set, gene symbols contained the chromosome name appended to the gene id. Therefore, after dimensionality reduction, we averaged the expression of genes with the same gene symbols but in different chromosomes to facilitate GO analysis. To identify potentially meaningful genes for trajectory analysis, we performed a PCA using 13,673 genes and selected genes with absolute loadings higher than a quantile of 0.975 in the first five principal components, resulting in 2550 genes to be analyzed regarding their regulation along multi-state trajectories. Cell cycle genes (GO:0007049) were removed prior to Gene Ontology analysis of cell-state transition-specific genes. Subpopulation 20 was selected as the starting population for trajectory identification because it is the most distant HSC subpopulation relative to all other subpopulations.

### Analysis of the BloodNet data set

We downloaded processed data from http://blood.stemcells.cam.ac.uk/single_cell_atlas.html (accession number: GSE81682). The data set made available by the authors was previously filtered to include only the most variable genes. To increase comparability with the previous study, we used the diffusion map coordinates generated by the authors in the original publication as a space of reduced dimensionality and built a kNN graph from the three-dimensional diffusion components available. We used *k* = 20 for subpopulation identification, to capture major subpopulations, and *k* = 10 for trajectory detection, as the kNN graph generated by *k* = 10 was already fully connected. This allowed the study of differentiation trajectories from HSPCs into three major lineages: erythroid, granulocyte-macrophage, and lymphoid. Cell cycle genes (GO:0007049) were removed prior to Gene Ontology analysis.

### Analysis of the human hematopoietic stem cell data set

We followed the procedure described in https://git.embl.de/velten/STEMNET/tree/master/vignettes to reproduce the analyses reported in Velten et al.^21^ (accession number: GSE75478). We used 12,281 genes for trajectory analysis and GRN reconstruction. We built a kNN graph from the low-dimensional embedding identified by STEMNET and set *k* = 13 for subpopulation identification, to capture major subpopulations, and *k* = 10 for trajectory detection, as the kNN graph generated by *k* = 10 was already fully connected.

### Analysis of myeloid progenitor data

We followed the procedure described in http://cole-trapnell-lab.github.io/monocle-release/Paul_dataset_analysis_final.html to apply Monocle 2 to analyze the data generated by Paul et al. and generated a processed data set that was used to compare CellRouter to Monocle 2, DPT, Wishbone, and Waterfall. Briefly, we downloaded scripts to reproduce the analysis in Paul et al. from http://compgenomics.weizmann.ac.il/tanay/?page_id=649. UMI counts were downloaded from GEO accession number GSE72857. A final data set of 3004 informative genes and 2699 cells (lymphoid cells were excluded) was used to compare these five algorithms. We applied Monocle 2 to this data set as described in the Monocle 2 tutorial and DPT as described in https://github.com/theislab/scAnalysisTutorial/blob/master/MARSseq_analysis_tutorial.ipynb. To run Wishbone, we used the top five principal components as input to perform the t-SNE analysis required by Wishbone and selected the diffusion components 1 and 3 as input. We used 150 waypoints. To run CellRouter in this data set, we selected *k* = 10 to build the kNN graph. Root cells were properly selected for each software (a cell in the common myeloid progenitors population), except for Waterfall, that does not have an option to define the starting point of the trajectory. In CellRouter, we selected the starting subpopulation 20 and a cell within this population is properly selected (as described in the section “Selecting sources and targets”).

### Re-analysis of time-course neutrophil differentiation

Bulk RNA-seq data profiling a time-course of neutrophil differentiation were downloaded from the GEO accession number GSE84874. Processed counts were publicly available. Then, we used the DESeq2 package to normalize counts by library size. Next, we averaged the normalized counts for each replicate (two per time point)) along the time-course of neutrophil differentiation. This data set was then used to validate dynamic expression patterns from the CellRouter trajectory from HSCs to neutrophils.

### Analysis of granulocyte and monocyte differentiation

We downloaded expression data, meta-data information, and a list of “guide genes” used in the original publication from https://www.synapse.org/#!Synapse:syn4975057/files/. In total, 382 wild-type cells were analyzed. We built a t-SNE map using 532 guide genes identified by iterative clustering in the original publication^13^. Then, we performed a PCA using all genes remaining after filtering out zero-variance genes (using 15,602 genes) and selected genes with absolute loadings higher than a quantile of 0.975 in the first five principal components, resulting in 3813 genes to be analyzed regarding their regulation along multi-state trajectories. We set *k* = 4 for subpopulation identification. Using a small *k* allows identification of a larger number of subpopulations, providing a refined subpopulation structure. However, this usually generates an unconnected graph. For trajectory identification, the graph needs to be connected or only transitions between subpopulations in the same connected component will be identified. Therefore, we set *k* = 15 to build a connected graph and identify multi-state differentiation trajectories, using the subpopulation structure identified using *k* = 4. Cell cycle genes (GO:0007049) were removed prior Gene Ontology analysis of cell-state transition-specific genes. Accession number: GSE70245.

### Analysis of mesoderm diversification

We downloaded processed data from http://gastrulation.stemcells.cam.ac.uk/scialdone2016. We used a list of the most variable genes across all single-cells for t-SNE analysis (list provided by the authors of the original publication^32^). To identify genes to be used for trajectory analysis, we combined genes with high absolute loadings from a PCA analysis with a list of about 1900 most variable genes identified by the authors of the original publication. We set *k* = 5 to identify subpopulations and *k*=15 to identify single-cell trajectories. Accession number: E-MTAB-4079.

### Detection of subpopulation-specific gene signatures

We identified subpopulation-specific gene signatures by comparing each subpopulation to all other subpopulations and computing a *p*value for the difference in mean expression between the subpopulation and cells in other subpopulations based on a binomial counting statistics^12^.

### Identification of genes regulated during differentiation

For each gene in each trajectory, we detect genes likely directly implicated in differentiation by ranking them by their correlation with the trajectory progression. For all data sets, we use the Spearman rank correlation coefficient to identify genes correlated or anti-correlated with the trajectory progression. Then, we consider genes above the 85% quantile as positively correlated with the trajectory progression (increasing expression during differentiation) and genes below the 15% quantile as anti-correlated with the trajectory progression (decreasing expression during differentiation). We identify more complex gene expression patterns by clustering expression trends into a user-defined number of clusters. Additional details are provided in the section “Analysis of gene expression dynamics”.

### Gene regulatory network reconstruction

We reconstructed GRN from single-cell transcriptomes for each data set. We used our previously developed algorithm, the CLR, especially the modified version published with CellNet to reconstruct GRNs for each data set. We hypothesize that the large-scale nature of single-cell data sets and the intrinsic population heterogeneity (including the presence of multiple cell types and states) would act as perturbations that could be explored by CLR to identify putative regulatory relationships.

### **In vitro** erythroid differentiation

Mobilized peripheral blood CD34+ stem/progenitor cells (AllCells) were obtained from three independent donors. Cells were expanded at 37 °C and 5% CO_2_ in StemSpan™ SFEM (StemCell Technologies) supplemented with the human recombinant cytokines 50 ng/ml SCF, 50 ng/ml Flt3L, 50 ng/ml TPO, 50 ng/ml IL-6, 10 ng/ml IL-3 (PeproTech) and 1% penicillin/streptomycin. Following 8 days of expansion, referred herein to as day 0 of differentiation, cells were moved to an erythroid differentiation medium based on Lee et al.^15^, consisting of Iscove’s modified Dulbecco’s medium with 2 mM glutamine, 15% FBS, 1% BSA, 500 μg/ml holo-Transferrin, 10 μg/ml insulin, 1% penicillin/streptomycin and the following stage-specific additions: 1 μM dexamethasone, 1 μM β-estradiol, 5 ng/ml IL-3, 100 ng/ml SCF and 6 U/ml EPO (days 0–4); 50 ng/ml SCF and 6 U/ml EPO (days 5–8); and 2 U/ml EPO (days 9–17).

### Cell staining

For flow cytometry, cells were stained with human CD71-PE (Clone-M-A712; BD Biosciences), human CD235a/Glycophorin A (11E4B-7–6; Beckman Coulter) and DAPI, and analyzed on a BD Fortessa cytometer. For histologic staining, cells were spun onto glass slides at 1000 rpm for 10 min and stained with May Grünewald for 12 min and Giemsa for 2 min. Images were acquired on Nikon Eclipse 90i with a Nikon Digital Imaging head.

### Gene expression analysis

Total RNA was extracted using the RNeasy Micro Kit and contaminating DNA was removed with the RNase free DNase kit (Qiagen). cDNA was synthesized from 200 ng of RNA using Maxima™ First Strand cDNA Synthesis Kit for RT-qPCR (Thermo Fisher Scientific). Real-time qPCR was performed using TaqMan Gene Expression Assays on QuantStudio 7 Flex. Each gene was run in triplicates and normalized to the housekeeping gene *GAPDH*.

### Data availability

The authors declare that all data supporting the findings of this study are available within the article and its supplementary information files or from the corresponding author upon reasonable request. No new data have been generated in this study. Data sets used in this study have already been deposited under accession codes: GSE76983 (for the mouse erythroblast/neutrophil differentiation data^12^), GSE84874 (for the bulk RNA-seq of mouse neutrophil differentiation data^14^), GSE81682 (for the BloodNet data^17^), GSE75478 (for the human HSPC data^21^), GSE72857 (for the mouse myeloid progenitors data^27^), GSE70245 (for the mixed-lineage states data, where only wild-type cells were analyzed^13^), and E-MTAB-4079 (for the mesoderm data, where only wild-type cells were analyzed^32^). Scripts to reproduce results in this paper (Supplementary Software 1–4) and the CellRouter source code (Supplementary Software 5) are available as Supplementary Software as well as through GitHub (https://github.com/edroaldo/cellrouter). Processed data are available through the CellRouter GitHub webpage.

## Electronic supplementary material

Supplementary Information

Description of Additional Supplementary Files

Supplementary Data 1

Supplementary Data 2

Supplementary Data 3

Supplementary Data 4

Supplementary Data 5

Supplementary Data 6

Supplementary Data 7

Supplementary Data 8

Supplementary Data 9

Supplementary Data 10

Supplementary Data 11

Supplementary Software 1

Supplementary Software 2

Supplementary Software 3

Supplementary Software 4

Supplementary Software 5
