# Supplementary material for: Reconstruction of complex single-cell trajectories using CellRouter
Source: Nat Commun. 2018 Mar 1;9:892. doi: 10.1038/s41467-018-03214-y (PMC5832860; doi:10.1038/s41467-018-03214-y)
Supplement: Supplementary file 1 — Supplementary Information [file 41467_2018_3214_MOESM1_ESM.pdf]

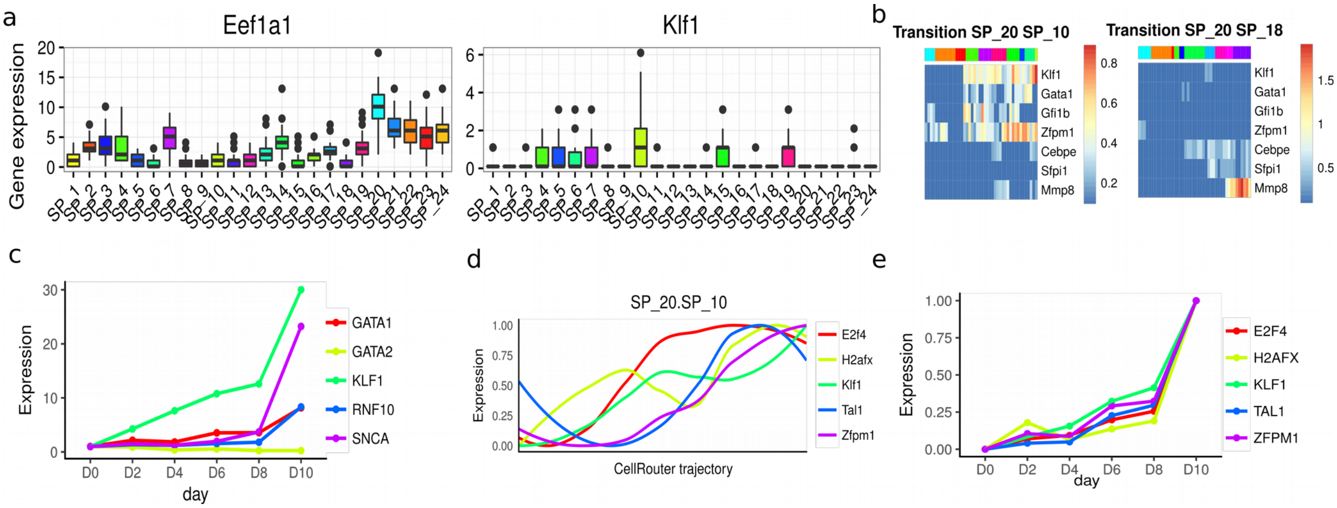

Supplementary Figure 1 Dynamics of cell-fate transitions to neutrophils and erythroblasts in the mouse bone marrow. **(a)** Expression levels of Eef1a1 across all subpopulations demonstrating higher expression levels in hematopoietic stem cell subpopulations and Klf1 expression across all subpopulations, demonstrating higher expression in subpopulation 10. **(b)** Positive and negative controls of erythrocyte and neutrophil differentiation. **(c)** qPCR of genes regulated during mouse and human erythrocyte differentiation (curves were not rescaled as in Figure 3e, bottom panel). **(d)** Transcription dynamics of transcription factors significantly upregulated in subpopulation 10. (**e**) qPCR validation along a time-course of human erythroid differentiation from CD34+ peripheral blood.

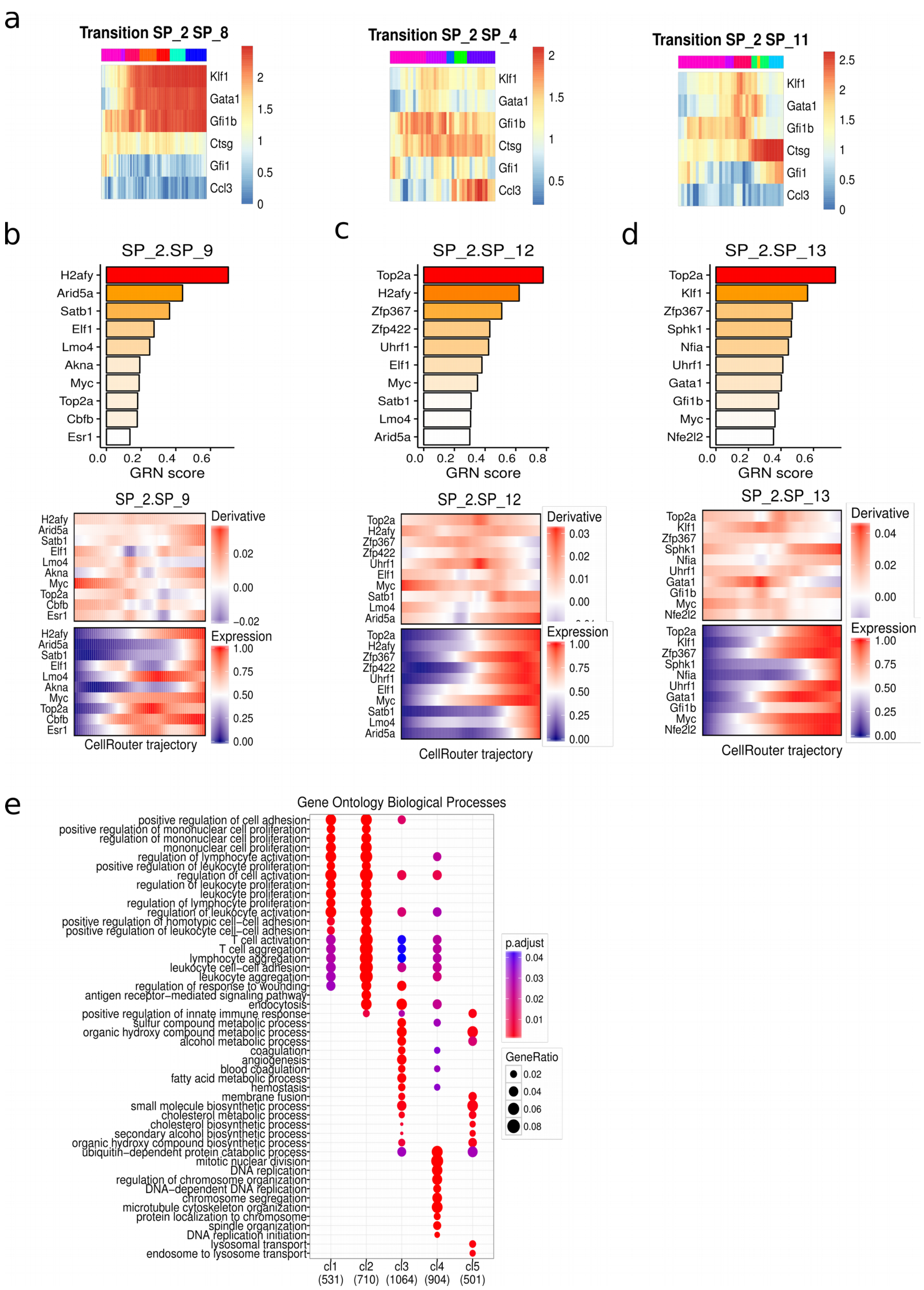
a
Transition SP_2 SP_8
Transition SP_2 SP_4
Transition SP_2 SP_11
Klf1
Gata1
Gfi1b
Ctsg
Gfi1
Ccl3
b
SP_2.SP_9
H2afy
Arid5a
Satb1
Elf1
Lmo4
Akna
Myc
Top2a
Cbfb
Esr1
GRN score
c
SP_2.SP_12
Top2a
H2afy
Zfp367
Zfp422
Uhrf1
Elf1
Myc
Satb1
Lmo4
Arid5a
d
SP_2.SP_13
Top2a
Klf1
Zfp367
Sphk1
Nfia
Uhrf1
Gata1
Gfi1b
Myc
Nfe2l2
Derivative
Expression
CellRouter trajectory
e
Gene Ontology Biological Processes
positive regulation of cell adhesion
positive regulation of mononuclear cell proliferation
regulation of mononuclear cell proliferation
mononuclear cell proliferation
regulation of lymphocyte activation
positive regulation of leukocyte proliferation
regulation of cell activation
regulation of leukocyte proliferation
leukocyte proliferation
regulation of lymphocyte proliferation
regulation of leukocyte activation
positive regulation of homotypic cell−cell adhesion
positive regulation of leukocyte cell−cell adhesion
T cell activation
T cell aggregation
lymphocyte aggregation
leukocyte cell−cell adhesion
leukocyte aggregation
regulation of response to wounding
antigen receptor−mediated signaling pathway
endocytosis
positive regulation of innate immune response
sulfur compound metabolic process
organic hydroxy compound metabolic process
alcohol metabolic process
coagulation
angiogenesis
blood coagulation
fatty acid metabolic process
hemostasis
membrane fusion
small molecule biosynthetic process
cholesterol metabolic process
cholesterol biosynthetic process
secondary alcohol biosynthetic process
organic hydroxy compound biosynthetic process
ubiquitin−dependent protein catabolic process
mitotic nuclear division
DNA replication
regulation of chromosome organization
DNA−dependent DNA replication
chromosome segregation
microtubule cytoskeleton organization
protein localization to chromosome
spindle organization
DNA replication initiation
lysosomal transport
endosome to lysosome transport
cl1 (531)
cl2 (710)
cl3 (1064)
cl4 (904)
cl5 (501)
p.adjust
GeneRatio

Supplementary Figure 2 Dynamics of transitions to intermediate subpopulations and transcriptional clusters during lymphoid development. **(a)** Positive and negative controls of each differentiation trajectory. **(b)** Predicted regulators of progenitor states of lymphoid, **(c)** granulocyte-macrophage and **(d)** erythroid differentiation from hematopoietic stem cells. Bottom panels show the transcriptional dynamics of predicted regulators of these transitions. **(e)** Gene Ontology analysis for genes in different transcriptional clusters during lymphoid progenitor differentiation.

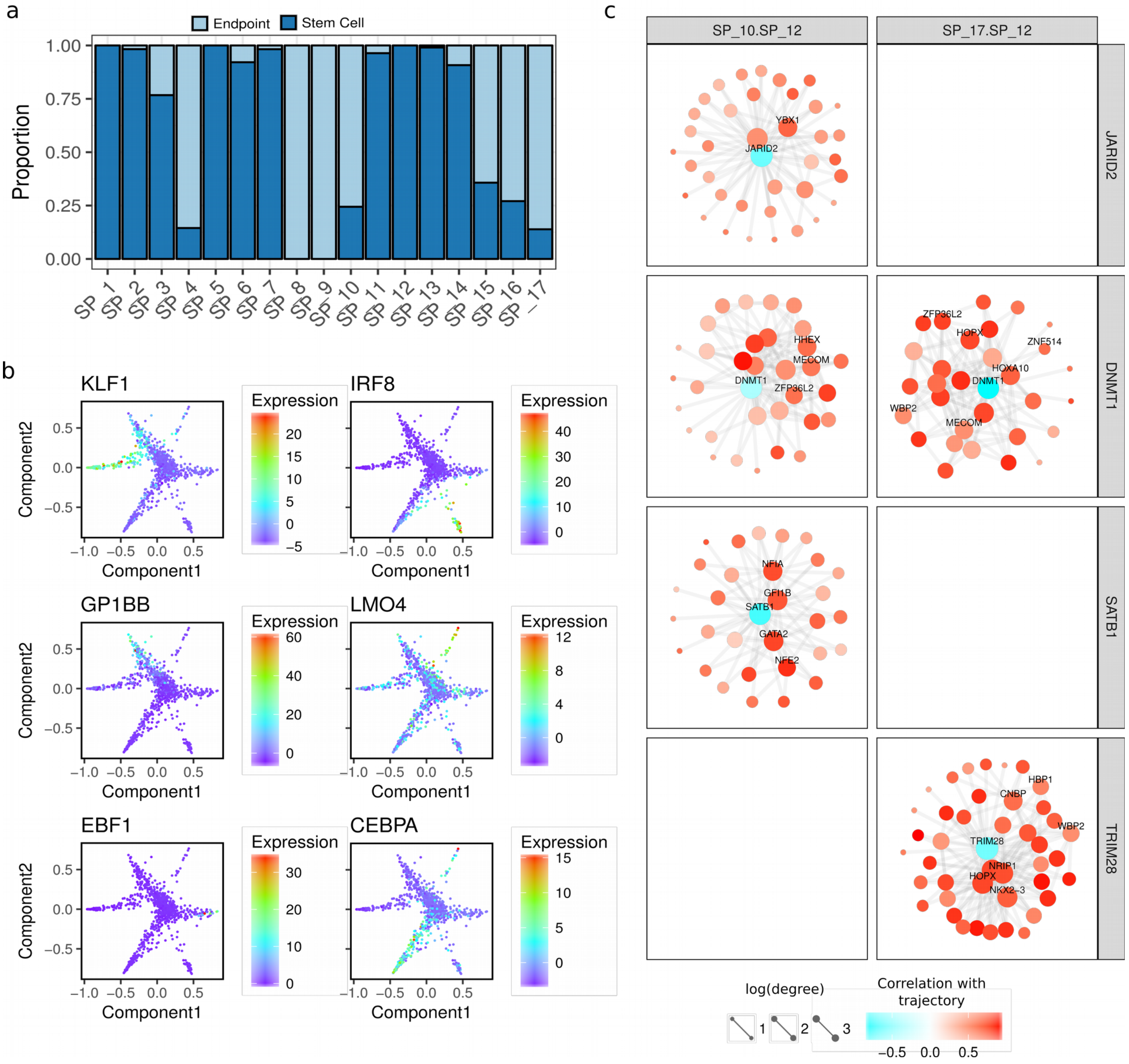

Supplementary Figure 3 Cell type annotation, differentiation markers and repressive epigenetic factors. (a) Annotation of cell types based on "Endpoint" or "Stem Cell", according to the original study. **(b)** STEMNET dimensionality reduction colored by expression of selected markers of each lineage. **(c)** Epigenetic factors predicted to suppress expression of HSPC genes.

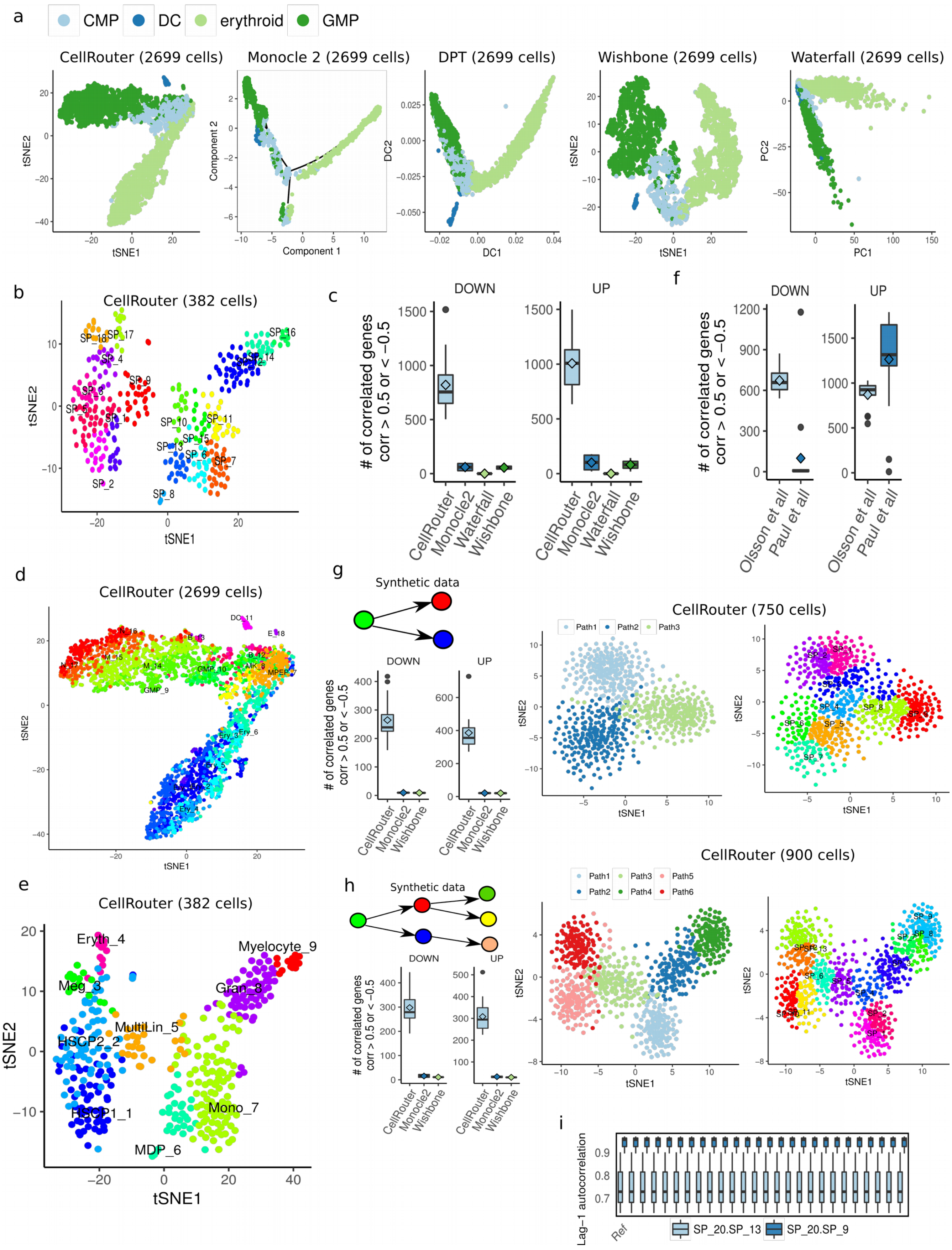
a
CMP
DC
erythroid
GMP
CellRouter (2699 cells)
Monocle 2 (2699 cells)
DPT (2699 cells)
Wishbone (2699 cells)
Waterfall (2699 cells)
tSNE1
tSNE2
Component 1
Component 2
DC1
DC2
PC1
PC2
b
CellRouter (382 cells)
c
DOWN
UP
# of correlated genes
corr > 0.5 or < −0.5
CellRouter
Monocle2
Waterfall
Wishbone
f
Olsson et all
Paul et all
d
CellRouter (2699 cells)
e
CellRouter (382 cells)
Eryth_4
Meg_3
MultiLin_5
HSCP2_2
HSCP1_1
MDP_6
Myelocyte_9
Gran_8
Mono_7
g
Synthetic data
CellRouter (750 cells)
Path1
Path2
Path3
h
Synthetic data
CellRouter (900 cells)
Path4
Path5
Path6
i
Lag−1 autocorrelation
Ref
SP_20.SP_13
SP_20.SP_9

Supplementary Figure 4 Comparison to other algorithms. (a) Dimensionality reduction maps generated by each trajectory identification algorithm annotated by cell types present in the myeloid progenitors dataset generated by Paul et al.[1] **(b)** CellRouter analysis of the dataset generated by Olsson et al.[2] **(c)** Number of genes significantly correlated to the trajectories identified by each algorithm in the dataset generated by Olsson et al.[2] **(d)** CellRouter analysis using t-SNE of the myeloid progenitor dataset using the clusters identified in the original publication by Paul et al.[1] **(e)** CellRouter analysis using t-SNE of the dataset generated by Olsson et al.[2] using the clusters identified by the authors in their original publication. **(f)** Comparison of the number of significantly correlated genes identified by CellRouter in the Paul et al. and Olsson et al. datasets[1,2]. **(g)** Left: Number of genes significantly correlated to the trajectories identified by each method using synthetic data representing one bifurcation event. Right: t-SNE maps and CellRouter analysis of this synthetic dataset. Path 1 represents the origin of the trajectories. **(h)** Number of genes significantly correlated to the trajectories identified by each method using synthetic data representing two bifurcation events. Right: t-SNE maps and CellRouter analysis of this synthetic dataset. Path 1 represents the origin of the trajectories. **(i)** Lag-1 autocorrelation of selected markers in Fig. 3e across 25 subsamplings of 80% of the original dataset compared to the autocorrelation coefficients calculated using the entire dataset (identified by "Ref" in the x-axis). As in Fig. 6e, differentiation trajectories from CMPs (subpopulation 20) to erythrocytes (subpopulation 13) or GMPs (subpopulation 9) were selected.

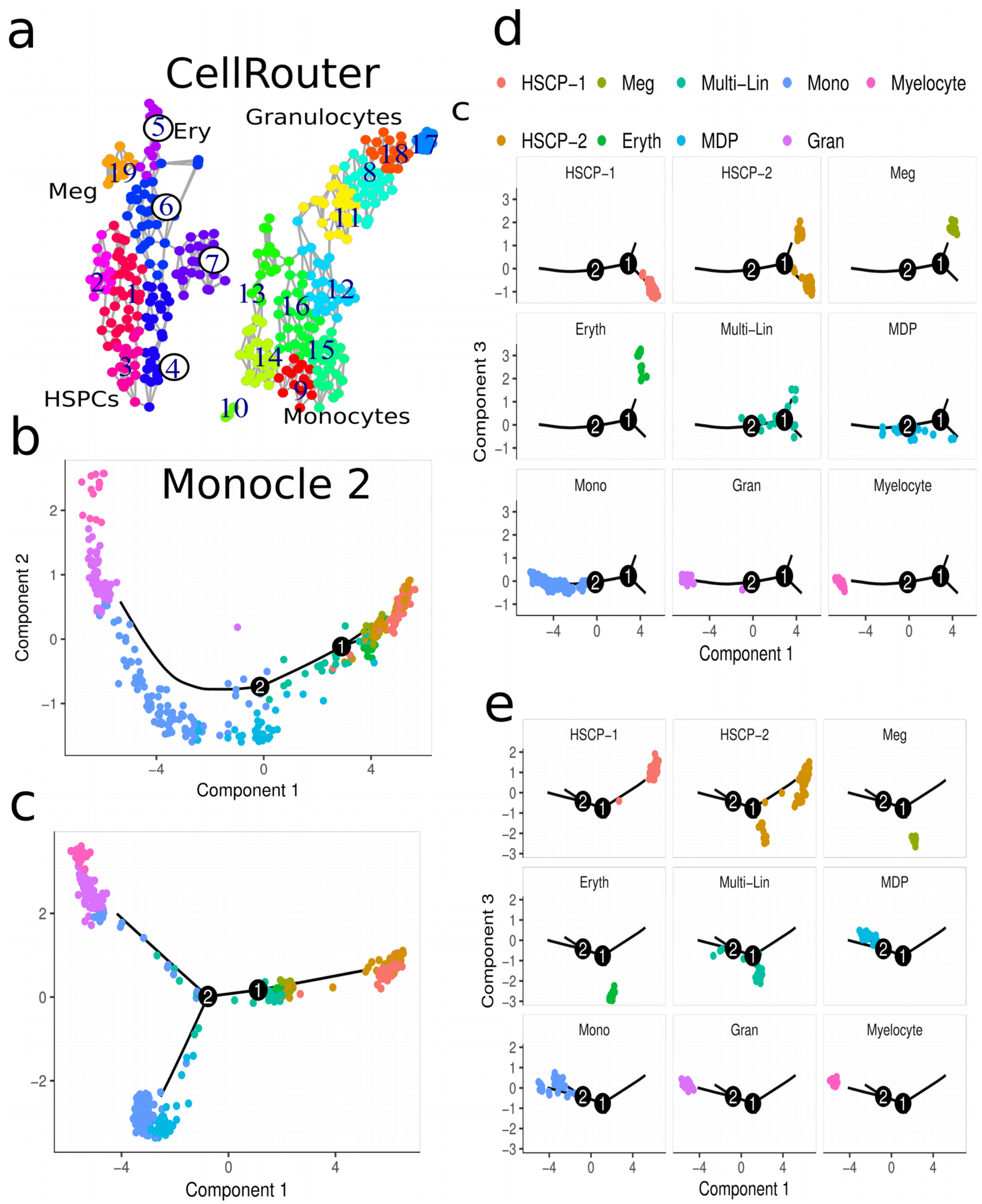

Supplementary Figure 5 Side-by-side comparison of CellRouter and Monocle 2 in the dataset generated by Olsson et al.[2] (a) CellRouter analysis with t-SNE generated using the most informative genes identified by Olsson at al[2]. and annotated by the major cell types present in the dataset. **(b)** Monocle 2 analysis using the same genes used by CellRouter for t-SNE dimensionality reduction. **(c)** Monocle 2 analysis using the top 1000 genes more differentially expressed between groups identified by Monocle 2. **(d)** Monocle 2 branching assignments and trajectories based on results from (b). **(e)** Monocle 2 branching assignments and trajectories based on results from (c). Note that megakaryocytes and erythrocytes are assigned to the same branch in both (d) and (e), indicating that Monocle cannot resolve this underrepresented bifurcation.

Supplementary Note 1: Minimum cost flow problem

We implemented the Ford-Fulkerson algorithm to solve the minimum cost flow problem in the k-nearest neighbor graph encoding Jaccard cell-cell similarities. We begin briefly introducing the concept of network flow and the maximum flow problem, which provides the framework to solve the minimum cost flow problem. When possible, we will make analogies related to the problem of identifying trajectories from single-cell data. Flow network algorithms provide a framework often used to optimize shipment of products between two locations. A flow network is a directed graph G(V,E) where V is a set of vertices and E a set of edges connecting vertices in V. A special node called source(s) produces units of a commodity that flow through the network to be consumed by a target node called sink or target (t). Each edge (u,v) has a flow f(u,v) that defines the number of units of the commodity that flows from u to v under constraints imposed to each edge, called the edge's capacity.

Maximum flow problem

Given a flow network, it is possible to compute the maximum flow over the network given the capacity constraints c(u,v) > 0 for all directed edges e=(u,v) in E. In other words, compute the largest amount that can flow from node s to node t given the capacity constraints in each edge. Starting from a feasible flow (a flow of zero is feasible), Ford-Fulkerson successfully finds an augmenting path from s to t to which more flow can be added. **Supplementary Fig. 6** shows a simple flow network that will be used to demonstrate how the algorithm works. Each edge is labeled as f/c to indicate the flow over the edge and its maximum capacity, respectively. Initially, the flow network has no flow (**Supplementary Fig. 6a**). Starting at node s, we augment the path s→2→4→t by transferring 2 units along this path (**Supplementary Fig. 6b**). Then, we augment s→1→3→t with 2 units (**Supplementary Fig. 6c**). The edge (3,t) is under-used. Then, we augment the path s→1→4→2→3→t with 1 unit and redirect flow from (2,4) over (2,3) (**Supplementary Fig. 6d**). Note that the capacities at the *s* and *t* nodes are full and additional flow through the network is not possible.

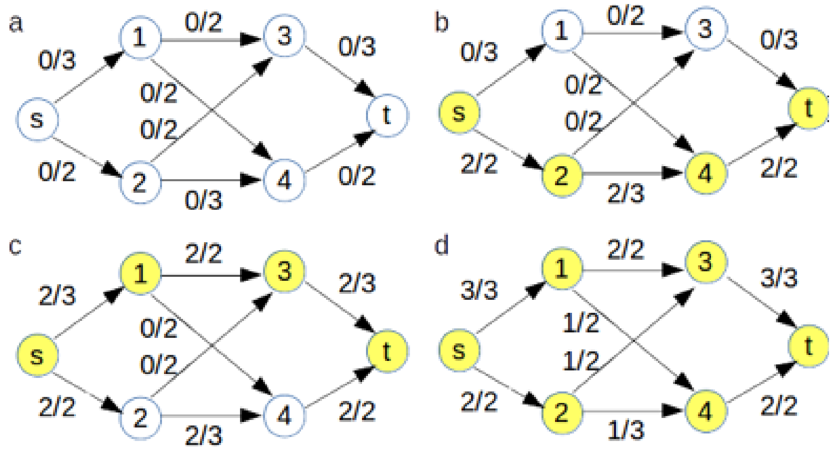

Supplementary Figure 6 Simple flow network to illustrate the maximum flow problem. (**a**) Starting flow network. (**b**) Select first path that maximizes flow. (**c**) Select next path that maximizes flow. (**d**) Edge capacities at the target node *t* are full and no more flow is possible.

Minimum cost flow problem

The minimum cost flow problem aims at obtaining the maximum flow through a network with the minimum cost. Therefore, in addition to the capacity, each edge also has a cost. Flow network algorithms provide an intuitive framework to develop trajectory detection algorithms from single-cell data. In this context, each node in the network is a single-cell and each edge represents connections between phenotypically related cell types or states. This information is encoded in a kNN graph. Given

a subpopulation structure, this framework allows the definition of many starting (stem cells) and target subpopulations (differentiated cells). The capacity is an upper bound for the similarity between any two cells. If two cells are highly similar, the edge connecting them will have a high capacity. We define the cost associated to each edge as the -log of the capacity such that minimizing the costs will give preference for high similarity paths in the kNN graph. As the total flow will be maximum at the lowest cost (similarity is being maximized), we reasoned that each path identified is optimal and they can be ranked by the total flow that they carry. CellRouter uses the top ranked path as the trajectory that describes the dynamic process taking place, such as differentiation.

The Ford-Fulkerson algorithm is based on a depth-first search to find augmenting paths through the flow network. The algorithm will find the maximum flow in the network regardless of the cost required. To find the augmenting path with lowest cost and then, solve the minimum cost flow problem, we implemented the Prim's algorithm, which is based on a priority queue to store the distance of each vertex in the network from the source vertex. Using this framework, CellRouter explores the subpopulation structure of single-cell datasets to find trajectories underlying the dynamic process taking place from source to target subpopulations without relying on any assumptions regarding branching processes or the number of branches in the cellular populations analyzed.

Allowing for multiple starting and target subpopulations

Cellular heterogeneity poses major challenges for single-cell analytics and current methods for trajectory identification are unlikely to perform well in this scenario because they are only able to find a trajectory between the most phenotypically distant cell states. In addition, these algorithms do not allow one to select a different starting or target population. For example, in stem cells, different starting populations could be biased to specific lineages and one would be interest to look at cell state transitions starting from this population towards each possible lineage, or a distinct maturation stage within that lineage branch. CellRouter allows one to identify a trajectory between any two given subpopulations. Given a list of starting subpopulations, CellRouter will automatically identify all possible trajectories to as many target subpopulations as present in the data. In addition, CellRouter takes as input coordinates in a space of reduced dimensionality. Therefore, any dimensionality reduction technique of preference can be used. None currently available algorithms can perform these tasks.

Applicability to large scale datasets

The most time consuming step in CellRouter is the dimensionality reduction step, which is not a limitation of CellRouter itself. The second more time consuming step is to fit smooth splines to the transcriptional dynamics of each gene (which is optional). This step depends on the number of genes used to perform the analysis, the number of subpopulations identified and the number of starting subpopulations selected. Larger number of genes and larger number of starting subpopulations will require more time to perform these steps. Importantly, all analysis performed in this paper were performed with a laptop computer with 12GB of RAM and none of them took longer than 30-60 minutes to be completed. Therefore, for larger single-cell transcriptomic datasets, with more than 5000 single-cells, the dimensionality reduction step could be performed in a super-computer environment and the remaining analysis could be performed local computer. CellRouter is a highly efficient algorithm to identify cell state transition trajectories in large and complex single-cell datasets.

Data embedding and visualization

While methods like Monocle 2, Diffusion pseudotime (DPT), Wishbone and Waterfall are restricted to specific dimensionality reduction techniques, such as reversed graph embedding (RGE, and variations of it), Diffusion Components (DC), t-SNE or PCA, respectively, for data visualization, CellRouter can

use coordinates generated by any dimensionality reduction technique to identify both trajectories and visualize cell-cell relationships, thereby providing an intuitive interpretation of the pseudotime.

Supplementary Note 2: Methodological comparisons to other algorithms

In this section we discuss the methodological differences of CellRouter, Monocle (1 and 2), Waterfall, Wishbone, Diffusion Pseudotime (DPT), StemID and Mpath to demonstrate the strengths of each method and how CellRouter introduces new concepts and also complements current single-cell trajectory analysis algorithms.

Working principle

Monocle 1[3] and Waterfall[4] use minimum-spanning trees in the Independent Component Analysis (ICA) embedding space and the Principal Component Analysis (PCA) space, respectively. Monocle 2[5] is based on a reversed graph embedding algorithm to learn a principal graph. The principal graph can be understood as a principal curve that passes through the “middle” of data with branches. This strategy allows reconstruction of complex trajectories with several branches by building an explicit tree through the data. DPT[6] is based on Diffusion Components (DC) and order cells based on geodesic distances calculated analytically. Wishbone[7] is based on heuristic approaches to learn a branching structure directly from the data by representing single-cells using a k-NN graph. Both DPT and Wishbone identify branches by analyzing patterns that diverge from a linear trajectory. However, Wishbone is limited to one bifurcation point and therefore, requires removal of cell types in branches not related to the differentiation process of interest. DPT can identify more than one branch but does not automatically determines how many “true” branches exist. These algorithms best identify trajectories between the most phenotypically distant cell types and are less robust in reconstructing trajectories towards intermediate stages of differentiation. This might impose restrictions to the experimental design where cellular populations have to be enriched for the cell types of interest or removed computationally. StemID[8] is designed for identification of a stem cell population in a mixture of cell types. It constructs a lineage tree by connecting cluster medoids in the embedding space, representing potential differentiation trajectories. Mpath[9] uses clustering to identify landmark clusters, which comprise cells mainly from one population. Then, builds a neighborhood network of landmarks in which edges connecting landmarks were weighted by the number of cells at the transitional stage. Low weighted edges are pruned, creating a network that represents cell-cell relationships.

We aimed at to develop an algorithm, CellRouter, that integrates subpopulation structure identification with cell-state transition trajectories. Moreover, it should scale with single-cell datasets containing random samplings of complex tissues such as the bone marrow, intestine, tumors or others, where the simultaneous identification of subpopulation structure (to identify rare and abundant cell types) and differentiation trajectories (to identify the dynamics of cell-state transitions) will be required. CellRouter takes a distinct approach by exploring the subpopulation structure of single-cell datasets to identify trajectories between any subpopulations, regardless of branching or maturation stage. CellRouter uses a network representation of cell-cell relationships learned from a low-dimensional embedding. This network, which is a kNN graph, encodes phenotypic relatedness and is used to determine subpopulation structure by identifying communities of densely connected cells. Then, CellRouter uses this network and subpopulation structure as a map of potential cell-fate transitions. Utilizing concepts from flow networks and solving the multi-source/multi-target minimum-cost flow problem to optimally connect cells in different locations of this map (subpopulations), CellRouter allows the study of expression dynamics in bifurcating or convergent differentiation paths in many different branches, including cell reprogramming trajectories. As it selects a subset of transitioning cells that are defined based on an optimization procedure, CellRouter trajectories are less noisy, with smoother gene

expression dynamics. The only parameter to be specified for a CellRouter analysis is *k*, the number of nearest neighbors to build a kNN graph from the single-cell data.

Supplementary Note 3: Application to other datasets

Transition-specific regulatory dynamics during granulocyte/monocyte differentiation

We applied CellRouter to single-cell RNA-seq data of murine hematopoietic stem/progenitor cells (HSPCs;lin-,Sca1+,c-Kit+ (LSKs)), common myeloid progenitors (CMPs), and granulocyte mononcyte progenitors (GMPs), and LK34+ cells (lin-,c-Kit+,CD34+). To increase comparability with the published analysis[2], we used the same gene set to perform dimensionality reduction with t-SNE[10]. We based the annotation of cell types on the original publication and subpopulation-specific gene expression signatures (**Supplementary Fig. 7a** and **8a, Supplementary Data 7**). CellRouter identified a refined subpopulation structure and two distinct and presumably bipotential subpopulations upstream of a predicted lineage bifurcation, one in an intermediate position between HSPCs and monocytes/granulocytes and another one preceding the megakaryocyte/erythrocyte divergence (**Supplementary Fig. 7a**).

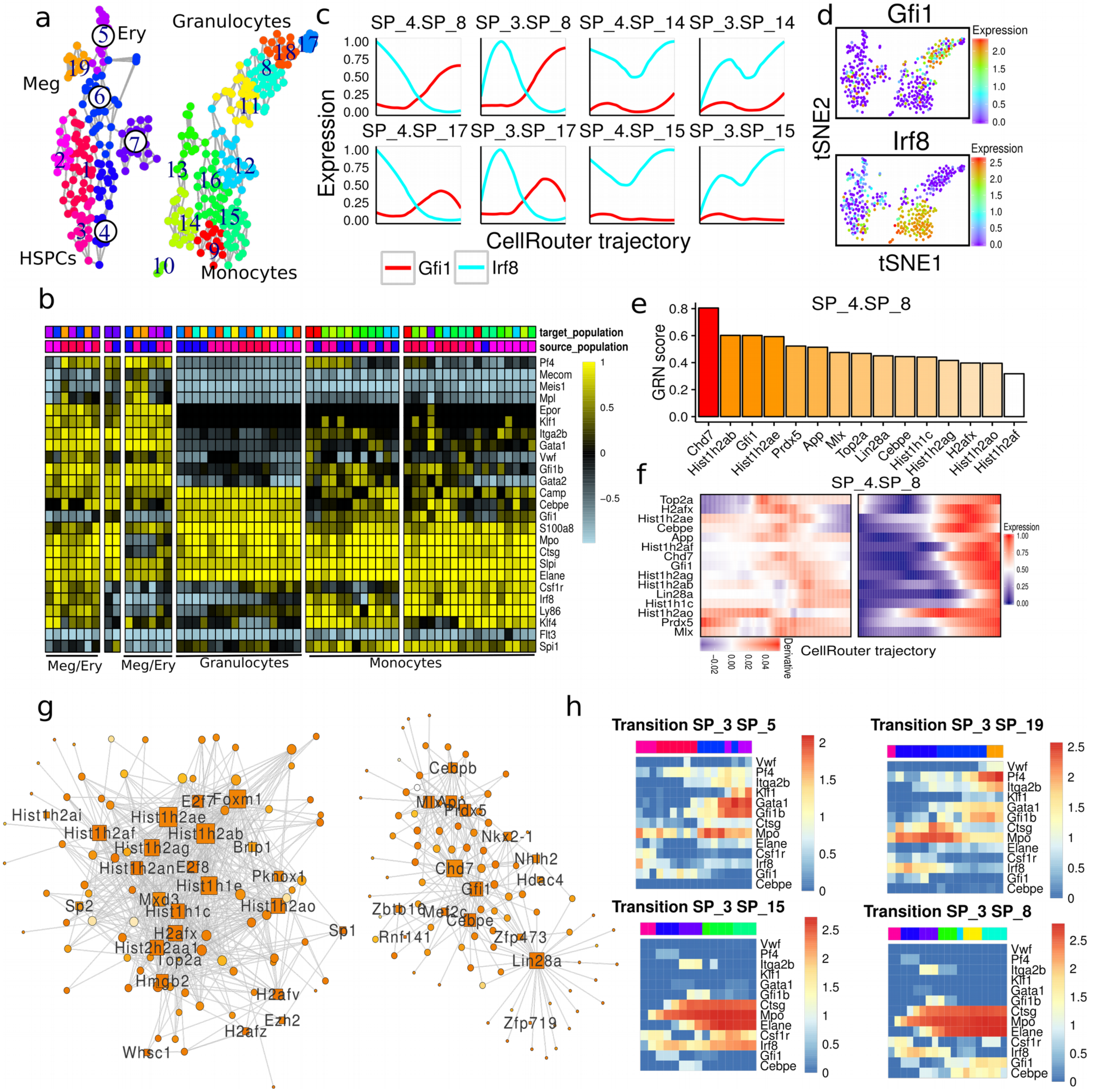

Supplementary Figure 7 Multi-lineage differentiation dynamics from HSPCs. **(a)** k-nearest neighbors graph built from t-SNE coordinates generated using guide genes identified in the original study. **(b)** Transcriptional dynamics of selected transcriptional factors and lineage specifying genes. **(c)** Expression trends of master regulators during differentiation from HSPCs to granulocytes and monocytes as well as myelocytes. **(d)** t-SNE map colored by expression of granulocyte (top panel) and monocyte (bottom panel) master regulators. **(e)** GRN score ranking the importance of transcriptional regulators for granulocyte development. **(f)** Gene expression dynamics along the trajectory showing where changes in expression occur as calculated by derivative analysis of expression curves along the trajectory (left panel) and the actual expression trend along the trajectory (right panel) for genes in (e). **(g)** Subnetwork centered around regulators identified in (e). **(h)** Positive and negative controls along selected differentiation trajectories.

Transcriptional dynamics of lineage-specific transcription factors and potential specifying genes selected by iterative clustering in the original publication confirmed the anti-correlated expression of Irf8 and Gfi1 in GMPs (**Supplementary Fig. 7b**) and revealed a potential early lineage priming in the

HSPCs towards monocyte differentiation (**Supplementary Fig. 7c,d**). Gfi1 and Irf8 are important pro-differentiation factors for granulocytes and monocytes, respectively. These analyses suggest that progenitor cells primed to monocytes intrinsically have low expression levels of transition-specific regulators of other lineages. However, state transitions to granulocytes might require concurrent up-regulation of Gfi1 and down-regulation of Irf8. As differentiation progresses, Gfi1 is downregulated in myelocytes (subpopulation 17), the most distant cell state in the granulocyte branch, suggesting that it is not required in late granulocyte differentiation (**Supplementary Fig. 7c,d**). Gene ontology (GO) analysis on genes upregulated during differentiation to representative subpopulations in each branch showed transition-specific expression dynamics to four different lineages, with enrichment for biological processes consistent with the respective cell types (**Supplementary Fig. 8b, Supplementary Data 8**). These analyses highlight the ability of CellRouter to illuminate transcriptional dynamics to subpopulations intermediate to stem cell and lineage-restricted mature subpopulations. Consistently, Gfi1 and Cepbe were among the top regulators during differentiation from the HSPC subpopulation 4 to the granulocyte subpopulation 8 (**Supplementary Fig. 7e**). To understand how these regulators are temporally related to each other, we computed the derivative of their expression dynamics along the trajectory from subpopulation 4 to 8. This analysis revealed that changes in Hist1h2ae, Cebpe, Prdx5 and Mlx occurred earlier than Gfi1, with coincident peak changes in Chd7 and Cebpe (**Supplementary Fig. 7f**). These genes formed a highly interconnected subnetwork, with a network module enriched with histone modifiers as well as interactions between Gfi1, Cepbe and Chd7 (**Supplementary Fig. 7g**).

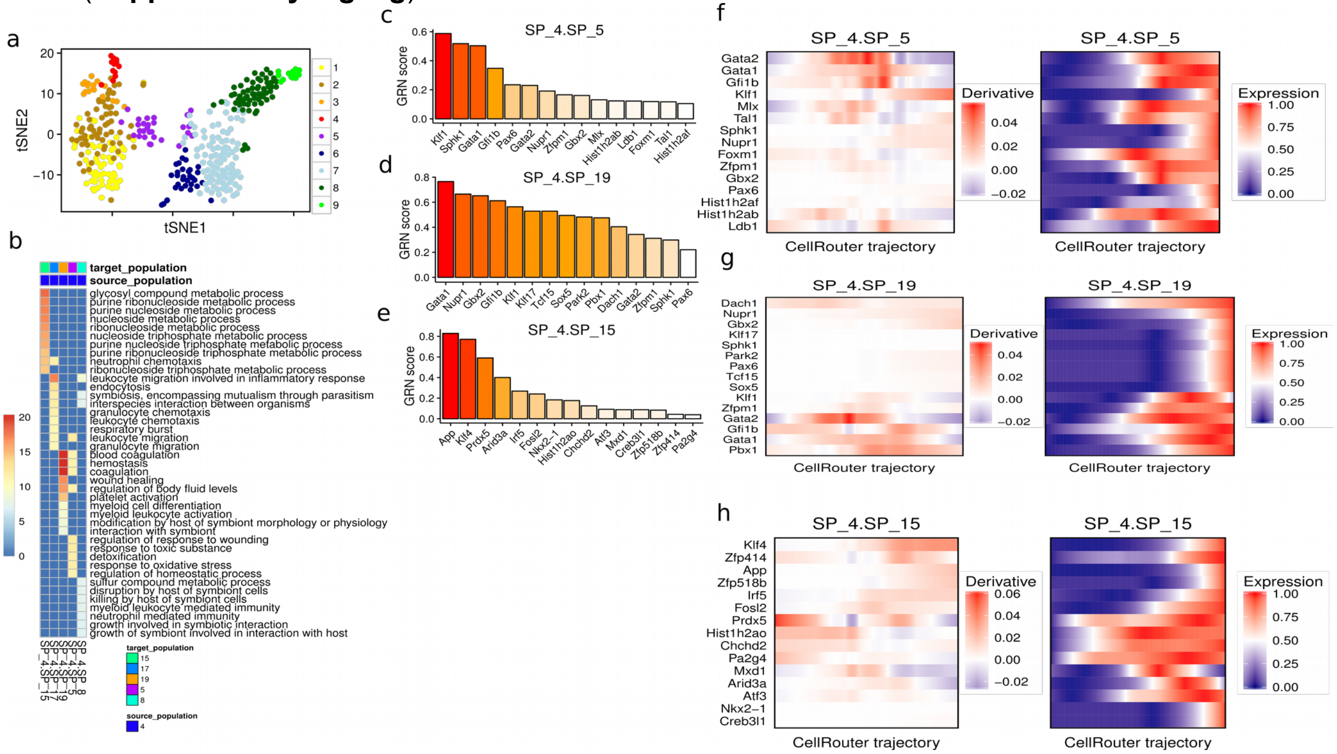

Supplementary Figure 8 Dynamics of lineage diversification to megakaryocyte/erythroid and granulocyte/monocyte linages. (a) t-SNE map colored by clusters identified in the original publication. **(b)** Gene Ontology analysis on transition-specific gene upregulated during differentiation to representative subpopulations in the erythroid/megakaryocyte and granulocyte/monocyte branches. **(c)** Predicted regulators of erythroid, **(d)** megakaryocyte and **(e)** monocyte differentiation. **(f)** Transcriptional dynamics of predicted regulators of erythroid **(g)** megakaryocyte and (i) monocyte differentiation.

Similar analysis on other cell state transitions also revealed transition-specific transcriptional regulators and their dynamics (**Supplementary Fig. 8c-h**). Transitions to megakaryocytes and erythrocytes share several genes, consistent with shared regulatory programs in these lineages[11], but

with distinct dynamics (**Supplementary Fig. 8f,g**). Consistent with its known biology, Gata2 was upregulated before Gata1 during erythroid differentiation while Klf1 up-regulation was observed in later stages (**Supplementary Fig. 8f**). These results showed that CellRouter can identify cell fate transition-specific expression dynamics and regulatory factors in intermediate and terminal cell states, in as many branches and cell states as present in the data.

Analysis of early mesoderm diversification towards primitive erythrocytes

We applied CellRouter to a time-course of mouse mesoderm diversification towards the hematopoietic system[12]. In this study, single-cell transcriptomes for the following mouse developmental stages were profiled: E6.5 (early gastrulation), E7.0 (primitive streak), E7.5 (neural plate) and E7.75 (head fold). Following subpopulation identification, we annotated cell types based on the original study and subpopulation-specific gene signatures (**Supplementary Fig. 9a, Supplementary Fig. 10a and Supplementary Data 9**). We then analyzed the differentiation trajectory from subpopulation 6 to subpopulation 15 to study the anterior-posterior axis of the primitive streak. Consistently, CellRouter identified genes known to be important for mesoderm development (**Supplementary Fig. 9b**). Msx2 is an important transcription factor for the epithelial-mesenchymal transition (EMT), which is essential during gastrulation. It has been reported as a mediator of BMP4-induced differentiation in human embryonic stem cells[13]. Interestingly, these genes showed early expression changes, with the highest changes coinciding with expression changes in Gata5, which is transiently upregulated and is required for heart and endoderm development in zebrafish[14] (**Supplementary Fig. 10b**). When starting the trajectory from subpopulation 11, not only have the GRN scores for shared regulators changed, but also new genes have appeared, such as Hoxb6 (**Supplementary Fig. 10c**, top panel). Consistently, Bmp4 and Podxl are upregulated in the posterior axis of the primitive streak, while Lefty2 and Tbx6 are downregulated (**Supplementary Fig. 9b**, bottom panel).

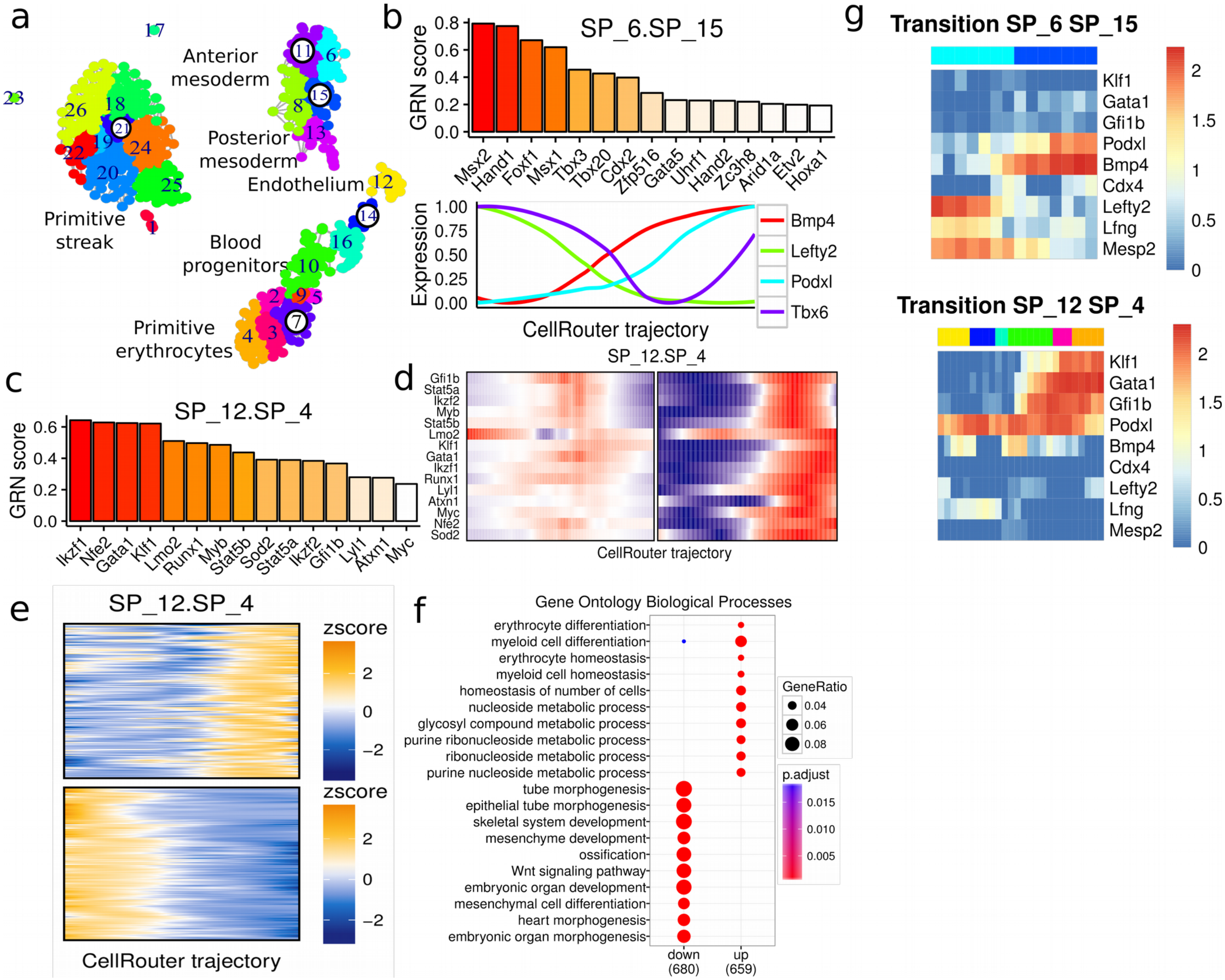

Supplementary Figure 9 Mesoderm diversification towards the hematopoietic system. (a) k-nearest neighbors graph built from t-SNE coordinates using the the most variable genes. **(b)** Predicted regulators of posterior mesoderm differentiation (top panel) and selected genes known to play a role in the anterior-posterior axis of mesodermal development (bottom panel). **(c)** Predicted regulators of cell fate transitions from endothelium to primitive erythrocytes. **(d)** Pseudo-temporal timing, where changes in predicted regulators from (c) happen along the trajectory to primitive erythrocytes (left panel) as well as their actual dynamics, being upregulated during differentiation (right panel). **(e)** Genes up- or downregulated during endothelium differentiation to blood lineages, and **(f)** their gene ontology enrichment. **(g)** Positive and negative controls of selected differentiation trajectories.

Gene Ontology analysis on genes downregulated during the mesoderm developmental trajectory revealed that genes in the anterior axis are related to gastrulation, somitogenesis, endoderm development and Notch signaling, consistent with a more anterior regulatory network[12] (populations 6-15). Conversely, enriched genes in the posterior populations are associated with BMP signaling and endothelium development (**Supplementary Fig. 10d , Supplementary Data 10**).

We next examined transcriptional programs activated during differentiation of endothelial cells (subpopulation 12) to primitive erythrocytes (subpopulation 4). Interestingly, known key factors in erythroid cell development have high GRN scores, consistent with the known biology (**Supplementary Fig. 9c**). Temporally, Stat5b, Lmo2 and Lyl1 showed early changes in expression, followed by Runx1, Nfe2 and Gif1b, then Gata1 and Klf1, which were more highly expressed towards the end of the trajectory towards blood (**Supplementary Fig. 9d**). Interestingly, Klf1 had a lower GRN score at

progenitor stages, supporting its importance in late stages of erythroid development (**Supplementary Fig. 10c**, bottom panel). Gene Ontology terms enriched along the blood differentiation trajectory were related to erythroid and myeloid differentiation as well as metabolic changes (**Supplementary Fig. 9e, f**). Processes downregulated included those associated with other mesoderm-derived tissue lineages, such as heart morphogenesis, consistent with specific commitment to the hematopoietic differentiation and repression of alternative lineages (**Supplementary Fig. 9e, f, Supplementary Data 11**). Positive and negative controls of mesoderm and erythroid development further demonstrated that CellRouter captures transition-specific gene expression dynamics (**Supplementary Fig. 9g**). Taken together, these data show that CellRouter identifies transcriptional regulators and their developmental timing during development.

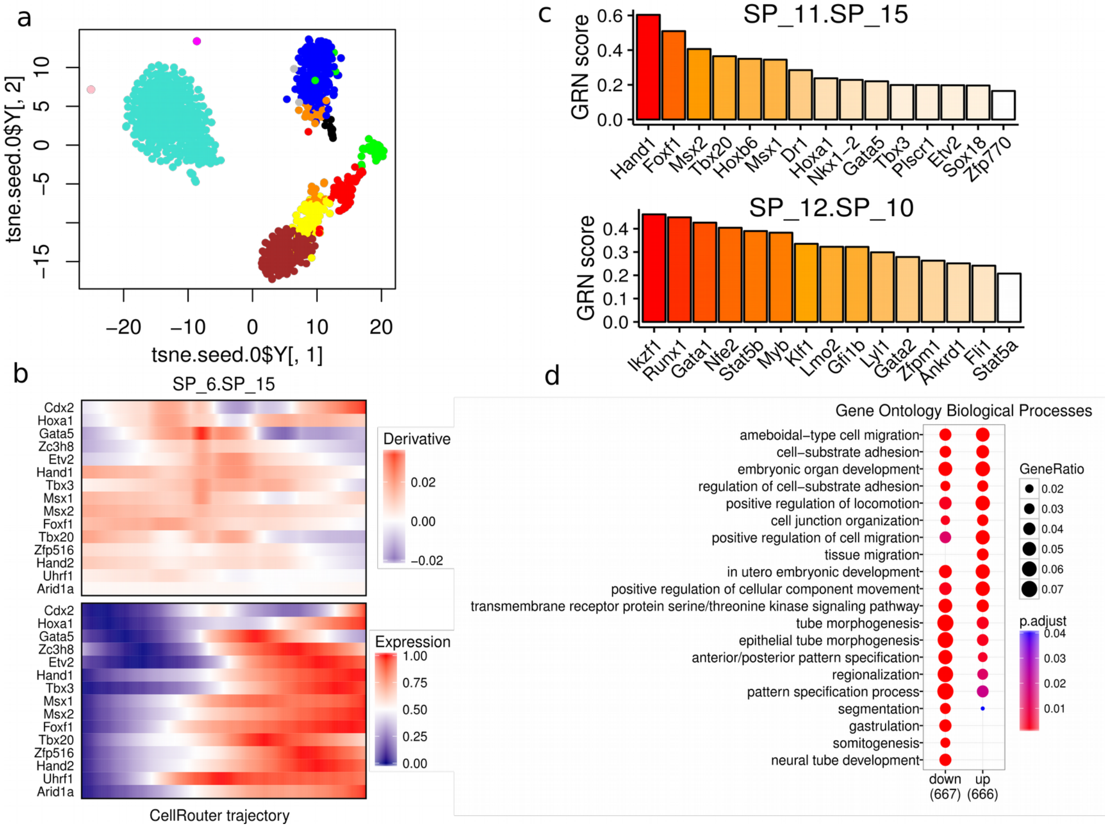

Supplementary Figure 10 Mesoderm diversification and cell fate transitions to erythroid progenitors. (a) t-SNE map colored by clusters identified in the original publication. **(b)** Transcriptional dynamics of predicted regulators of anterior-posterior mesoderm development. **(c)** Predicted regulators of mesoderm development using the mesoderm subpopulation 11 as the starting subpopulation for trajectory identification (top panel) and to erythrocyte progenitors (subpopulation 10). **(d)** Gene Ontology analysis on genes up- or downregulated along the mesoderm developmental trajectory.

Supplementary Note 4: dimensionality reduction and convergent differentiation

Effect of dimensionality reduction

We tested how the choice of dimensionality reduction technique affects trajectories identified by CellRouter. First, we reanalyzed the dataset presented in **Fig. 2** using Principal Component Analysis (PCA) and annotated the major branches based on marker gene expression (**Supplementary Fig.**

11a,b). These data demonstrate that CellRouter performs similarly when applied with PCA or t-SNE, identifying similar regulators of each cell-state transition as well as similar kinetic patterns (**Supplementary Fig. 11c,d**). We extended this analysis to t-SNE, PCA and Diffusion Components (DC) and applied CellRouter to a myeloid progenitor dataset generated by Paul et al.[1] (**Supplementary Fig. 12a-c**). Overall, CellRouter identified a substantial number of correlated genes, which expectedly varied across dimensionality reduction techniques (**Supplementary Fig. 12d**). We also performed a quantitative comparison of the gene expression dynamics of selected markers of differentiation in the erythrocyte (Klf1, Car2 and Cite4) and GMP (Mpo, Ctsg and Prtn3) branches by calculating the lag-1 autocorrelation of these genes along the corresponding trajectories (**Supplementary Fig. 12e,f**). The higher the autocorrelation, smoother gene expression dynamics is based on ordering of single-cells along the trajectory. This analysis showed that the dynamics reconstructed by CellRouter is very consistent across dimensionality reduction techniques.

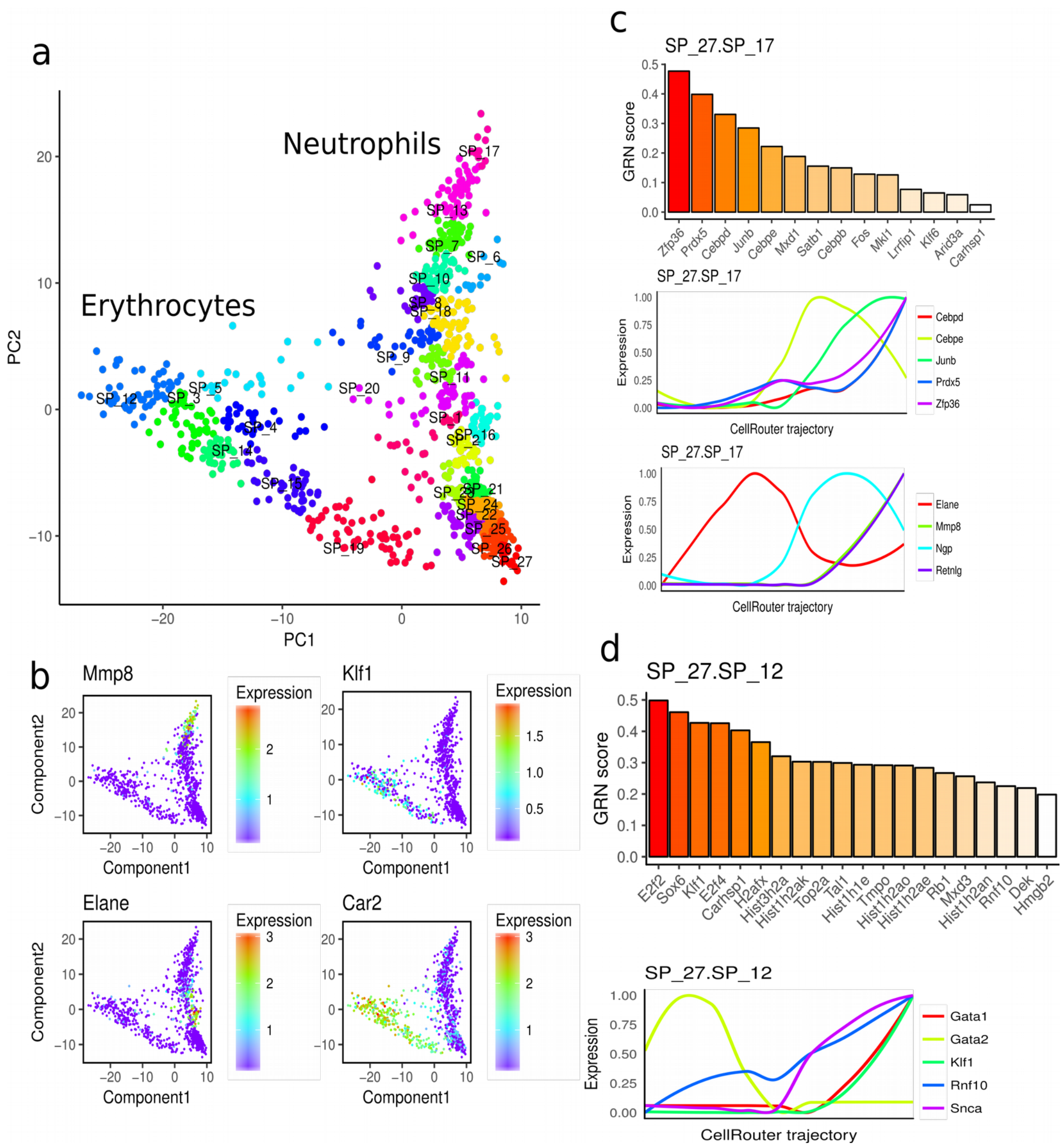

Supplementary Figure 11 Analysis of the mouse bone marrow dataset discussed in Figure 1 using Principal Component Analysis (PCA). (a) CellRouter analysis using PCA for dimensionality reduction. **(b)** Expression of marker genes to identify source subpopulation(s). **(c)** top panel: top predicted regulators of neutrophil differentiation, middle: gene expression dynamics of top five predicted regulators of neutrophil differentiation, bottom: gene expression dynamics of selected genes known to be important for different stages of neutrophil differentiation. **(d)** top panel: predicted regulators of erythrocyte differentiation (subpopulation 12 express highest Klf1 levels), bottom: gene expression dynamics of genes known to be important for erythrocyte differentiation and also potential new genes identified by CellRouter.

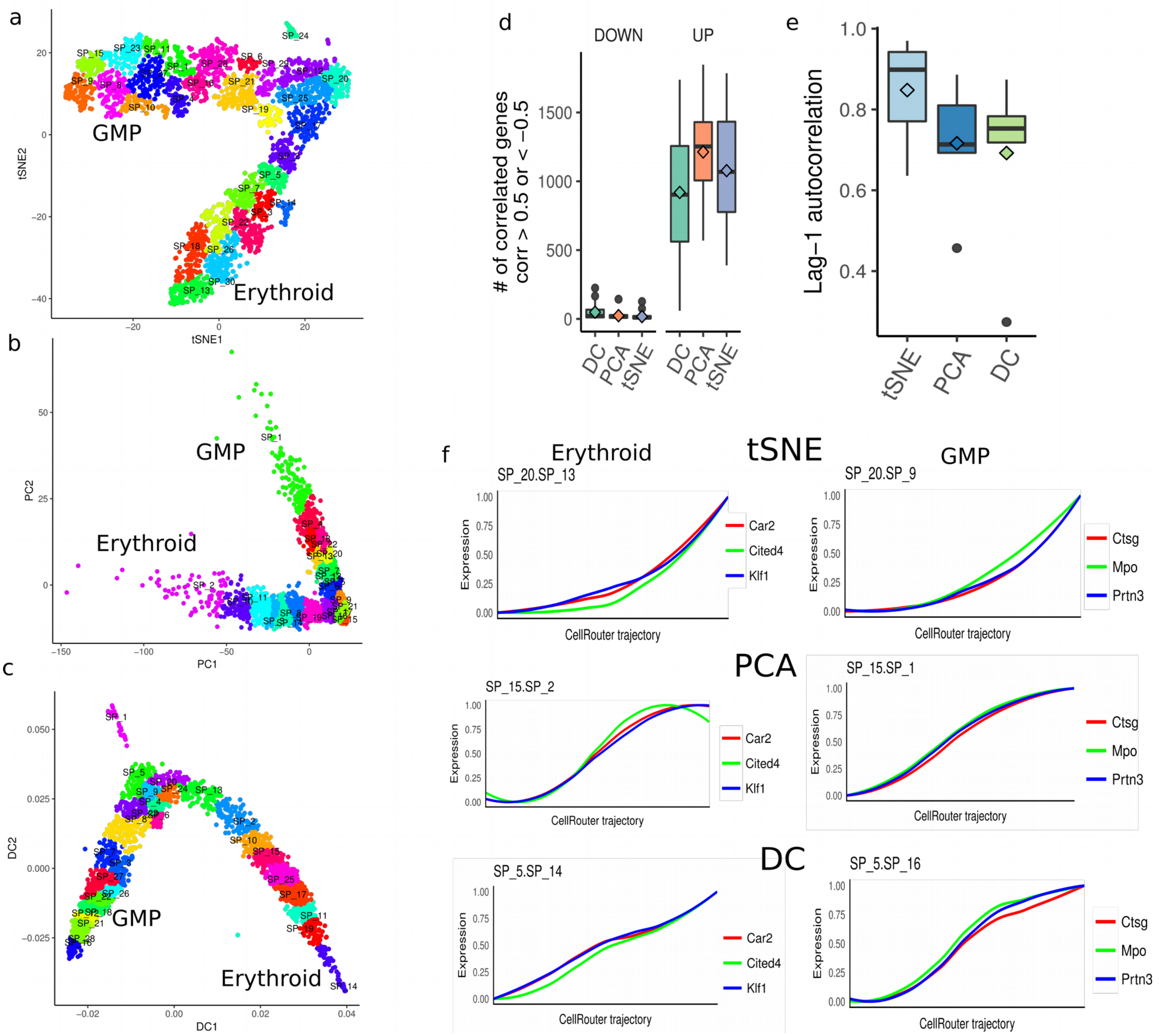

Supplementary Figure 12 Testing CellRouter with different dimensionality reduction techniques. (a) CellRouter analysis using t-SNE for dimensionality reduction. **(b)** CellRouter analysis using Principal Component Analysis (PCA) for dimensionality reduction. **(c)** CellRouter analysis using diffusion components (DC) for dimensionality reduction. **(d)** Number of significantly correlated genes identified by using DC, PCA or t-SNE. **(e)** Lag-1 autocorrelation of selected marker genes (Fig. 3e) in the GMP and erythrocyte differentiation trajectories. **(f)** Kinetic trends of selected marker genes (Fig. 3e) along the erythrocyte and GMP trajectories.

Convergent differentiation paths

We also assessed whether CellRouter can identify branch-specific gene expression dynamics in convergent differentiation paths, or to dedifferentiate/reprogram mature cell types to progenitor/stem cell states, as during cell reprogramming or cell fate engineering. First, we reversed the directionality of cell fate transitions in the myeloid progenitor dataset, identifying dedifferentiation trajectories from subpopulation 13 (in the erythrocyte branch) or subpopulation 9 (in the GMP branch), towards CMPs (subpopulation 20) (**Fig. 6b**). To compare the ability to perform similarly when identifying trajectories in both directions, we reasoned that genes upregulated during differentiation should be downregulated during dedifferentiation. Indeed, there is a high overlap of these gene sets, demonstrating a similar performance whether trajectories are reconstructed from stem cells to mature cell types or vice-versa (**Supplementary Fig. 13a**).

Moreover, we generated a synthetic dataset where two progenitor states converge to the same mature cell type, as during pDC development[15] (**Supplementary Fig. 13b**). We applied CellRouter to identify transitions from subpopulation 7 or 11 to subpopulation 1, simulating a convergent differentiation path (**Supplementary Fig. 13c**). Then, we asked whether CellRouter can identify transition-specific gene expression dynamics by evaluating how expression of genes dynamically regulated during a particular transition change in the other transition, where no pattern should be observed (**Supplementary Fig. 13d**). This analysis demonstrated that CellRouter can capture transition-specific genes in convergent differentiation paths.

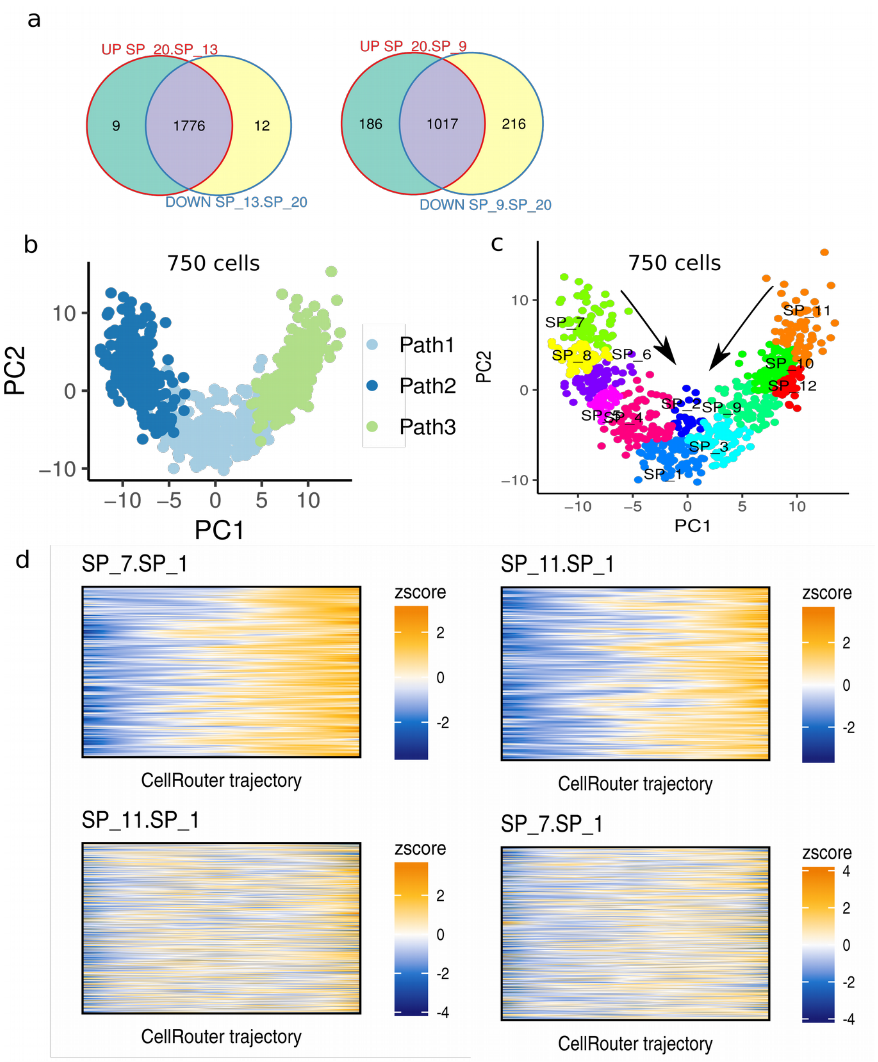

Supplementary Figure 13 Testing the effect of reversing the directionality of differentiation trajectories and convergent differentiation paths. (a) Overlap of genes upregulated from common myeloid progenitors (CMPs, subpopulation 20) to erythrocytes (subpopulation 13) or granulocyte/monocyte progenitors (GMPs) with genes downregulated when the trajectory is reversed. **(b)** Synthetic dataset representing a convergent differentiation path. Paths 2 and 3 are the starting points while path 1 is the end point for trajectory identification. **(c)** CellRouter analysis of the synthetic dataset in (b) selecting subpopulations 7 and 11 as the starting subpopulations that ultimately converge to subpopulation 1. **(d)** Transition specific gene expression dynamics from subpopulation 7 to 1 and from subpopulation 11 to 1.

Supplementary Method

Pseudocode for the CellRouter algorithm

Inputs

X: Dataset of m genes and n cells

L: Low dimensional embedding of dataset X generated by an user-preferred dimensionality reduction technique: PCA, t-SNE, Diffusion Components, Independent Component Analysis and so on.

GRN: gene regulatory network reconstructed from the single-cell expression data.

k: number of nearest neighbors

s: source subpopulation (for example, stem cells)

t: target subpopulations, automatically determined based on s. However, users can also provide a list of target subpopulations they are more interested in.

Algorithm

1. Construct a k-NN graph G using Euclidean distance in the embedded space L. Each cell is a node in the graph and a cell is connected by distance-based weighted edges to its k nearest neighbors.

2. Transform the k-NN graph G to contain similarity based-weighted edges using network similarity metrics, such as the Jaccard index.

3. Apply a community detection algorithm that maximize modularity to identify communities of densely connected cells, which we then call subpopulations.

4. Determine the source subpopulation(s) which will be used as starting point(s) for trajectory identification to all other subpopulations identified in Step 3.

5. Automatically define all subpopulations other than the source subpopulation(s) as target subpopulations. Or, users can also provide a list of target subpopulations that they want to study.

6. Identify the asource and target cell in each source and target subpopulation by selecting the first and last cell in the longest path from the source to the target subpopulation. The first cell in the path is defined as source. The last cell in the path is defined as target. This procedure is repeated for each pairwise transition trajectory.

7. Apply the flow network algorithm (the solves the minimum-cost flow problem) to precisely identify all possible paths connecting the source to the target subpopulations.

8. Rank these paths based on their total flow normalized by the length of the path and select the top ranked one as a representative differentiation trajectory.

9. Apply downstream analytics implemented in CellRouter to identify genes dynamically regulated during differentiation, temporal relationships between genes along differentiation, transition-specific

expression patterns and prioritization of potential regulators of cell fate transitions. Gene ontology and pathway enrichment analysis are also built-in functions in CellRouter.

Output

T: multi-state transition trajectories

G: genes dynamically regulated along each trajectory

C: cluster of gene expression kinetics (waves of transcriptional regulation along each trajectory)

R: regulators of cell fate transitions

E: enrichment analyses using gene ontology and reactome pathways

Supplementary references

1. Paul, F. *et al.* Transcriptional Heterogeneity and Lineage Commitment in Myeloid Progenitors. *Cell* **163,** 1663–1677 (2015).
2. Olsson, A. *et al.* Single-cell analysis of mixed-lineage states leading to a binary cell fate choice. *Nature* **537,** 698–702 (2016).
3. Trapnell, C. *et al.* The dynamics and regulators of cell fate decisions are revealed by pseudotemporal ordering of single cells. *Nat. Biotechnol.* **32,** 381–6 (2014).
4. Shin, J. *et al.* Single-Cell RNA-Seq with Waterfall Reveals Molecular Cascades underlying Adult Neurogenesis. *Cell Stem Cell* **17,** 360–372 (2015).
5. Qiu, X. *et al.* Single-cell mRNA quantification and differential analysis with Census. *Nat. Methods* (2017). doi:10.1038/nmeth.4150
6. Haghverdi, L., Büttner, M., Wolf, F. A., Buettner, F. & Theis, F. J. Diffusion pseudotime robustly reconstructs lineage branching. *Nat. Methods* **13,** 845–848 (2016).
7. Setty, M. *et al.* Wishbone identifies bifurcating developmental trajectories from single-cell data. *Nat. Biotechnol.* **34,** 1–14 (2016).
8. Grün, D. *et al.* De Novo Prediction of Stem Cell Identity using Single-Cell Transcriptome Data. *Cell Stem Cell* **19,** 266–277 (2016).
9. Chen, J., Schlitzer, A., Chakarov, S., Ginhoux, F. & Poidinger, M. Mpath maps multi-branching single-cell trajectories revealing progenitor cell progression during development. *Nat. Commun.* **7,** 11988 (2016).
10. Van Der Maaten, L. J. P. & Hinton, G. E. Visualizing high-dimensional data using t-sne. *J. Mach. Learn. Res.* **9,** 2579–2605 (2008).
11. Doré, L. C. & Crispino, J. D. Transcription factor networks in erythroid cell and megakaryocyte development. *Blood* **118,** 231–239 (2011).

12. Scialdone, A. *et al.* Resolving early mesoderm diversification through single-cell expression profiling. *Nature* **535,** 4–6 (2016).

13. Richter, A. *et al.* BMP4 promotes EMT and mesodermal commitment in human embryonic stem cells via SLUG and MSX2. *Stem Cells* **32,** 636–648 (2014).

14. Reiter, J. F. *et al.* Gata5 is required for the development of the heart and endoderm in zebrafish. *Genes Dev.* **13,** 2983–2995 (1999).

15. Sathe, P., Vremec, D., Wu, L., Corcoran, L. & Shortman, K. Convergent differentiation: Myeloid and lymphoid pathways to murine plasmacytoid dendritic cells. *Blood* **121,** 11–19 (2013).
