# Supplementary material for: Reconstruction of complex single-cell trajectories using CellRouter
Source: Nat Commun. 2018 Mar 1;9:892. doi: 10.1038/s41467-018-03214-y (PMC5832860; doi:10.1038/s41467-018-03214-y)
Supplement: Supplementary file 2 — Description of Additional Supplementary Files [file 41467_2018_3214_MOESM2_ESM.docx]

**Description of Additional Supplementary Files**

File Name: Supplementary Data 1

Description: Subpopulation-specific gene signatures in the mouse bone marrow.

File Name: Supplementary Data 2

Description: Gene ontology analysis of genes dynamically regulated along selected cell-state transitions from hematopoietic stem and progenitor cells to erythroblasts, neutrophils and intermediate states.

File Name: Supplementary Data 3

Description: Subpopulation-specific gene signatures in the BloodNet dataset.

File Name: Supplementary Data 4

Description: Gene ontology analysis of genes dynamically regulated multi-lineage differentiation from hematopoietic stem and progenitor cells.

File Name: Supplementary Data 5

Description: Gene ontology analysis of genes with complex expression kinetics during lymphoid differentiation.

File Name: Supplementary Data 6

Description: Libraries of transcription factors used in prior publications to convert a starting cell population to HSPCs.

File Name: Supplementary Data 7

Description: Subpopulation-specific gene signatures during erythroid, megakaryocyte, monocyte and granulocyte differentiation from hematopoietic stem and progenitor cells.

File Name: Supplementary Data 8

Description: Gene ontology analysis of genes dynamically regulated along selected cell-state transitions from hematopoietic stem and progenitor cells to megakaryocytes, erythrocytes, granulocytes and monocytes.

File Name: Supplementary Data 9

Description: Subpopulation-specific gene signatures during mesoderm diversification.

File Name: Supplementary Data 10

Description: Gene ontology analysis of genes dynamically up- or downregulated during mesoderm differentiation.

File Name: Supplementary Data 11

Description: Gene ontology analysis of genes dynamically up- or downregulated during blood differentiation.

File Name: Supplementary Software 1

Description: Script containing step-by-step instructions to reconstruct single-cell trajectories during erythroblast and neutrophil differentiation

File Name: Supplementary Software 2

Description: Script containing step-by-step instructions to reconstruct multi-lineage single-cell trajectories in the BloodNet dataset

File Name: Supplementary Software 3

Description: Script containing step-by-step instructions to reconstruct multi-lineange differentiation from human hematopoietic stem cells

File Name: Supplementary Software 4

Description: Script containing step-by-step instructions to reconstruct cell reprogramming trajectories from B-cells and monocyte/dendritic cells to hematopoietic stem and progenitor cells

File Name: Supplementary Software 5

Description: CellRouter main class, containing functions to reconstruct single-cell trajectories using CellRouter
